# Hepatocyte PIEZO1 Negatively Regulates Lipogenesis and Ameliorates MASLD by Sensing Membrane Tension and Activating AMPK

**DOI:** 10.1002/advs.202515847

**Published:** 2026-04-03

**Authors:** Hui Chen, Qimeng Wang, Ke Yang, Qinghai Lian, Xuyun Peng, Zhiyong Gong, Xinyi Zhao, Yong Wu, Tian Tao, Siqi Xu, Yifan Chen, Xiaoyan Tang, Jinghui Guo, Geyang Xu, Qi Zhang

**Affiliations:** ^1^ Biotherapy Center The Third Affiliated Hospital Sun Yat‐Sen University Guangzhou Guangdong China; ^2^ Cell‐gene Therapy Translational Medicine Research Center The Third Affiliated Hospital Sun Yat‐Sen University Guangzhou Guangdong China; ^3^ Guangdong Key Laboratory of Liver Disease Research The Third Affiliated Hospital Sun Yat‐sen University Guangzhou China; ^4^ Department of Physiology School of Medicine Jinan University Guangzhou China; ^5^ School of Medicine The Chinese University of Hong Kong Shenzhen Guangdong China

**Keywords:** AMPK, de novo lipogenesis, MASLD, membrane tension, PIEZO1

## Abstract

Liver is a central organ for lipid metabolism. Disruption of lipid homeostasis leads to lipid accumulation in hepatocytes, which is a feature of metabolic dysfunction‐associated steatotic liver disease (MASLD). Mechanical force and mechanosensitive proteins have been found to play a crucial role in energy metabolism. However, their role in hepatic lipid metabolism remains unclear. In this study, mechanosensitive ion channel PIEZO1 is detected in hepatocytes, and downregulated in the liver of MASLD patients and high‐fat diet (HFD)‐induced MASLD mouse model. Under HFD feeding, mice with hepatocyte‐specific *Piezo1* deletion exhibit severer triglyceride accumulation, upregulation of de novo lipogenesis genes, and decreased phosphorylation of AMPK and RAPTOR in the liver. In contrast, injection of PIEZO1 activator Yoda1 alleviates triglyceride accumulation, downregulates lipogenesis genes and enhances phosphorylation of AMPK and RAPTOR in HFD‐fed C57BL/6 mice. Knockdown of *PIEZO1* in HepG2 leads to upregulation of lipogenesis genes and impairs AMPK‐RAPTOR pathway, while Yoda1 or hypotonic treatment do the reverse. The effects of *PIEZO1* knockdown and Yoda1 treatment can be abolished by AMPK activator and CaMKK2/AMPK inhibitors, respectively. These findings suggest that PIEZO1 can respond to changes in membrane tension and activate AMPK, thereby inhibiting lipogenesis and maintaining lipid homeostasis.

## Graphical abstract

Hepatocytes are subjected to increased membrane tension along with lipid accumulation. Loss of PIEZO1 exacerbates high‐fat diet‐induced MASLD in mice. Activation of PIEZO1 alleviates high‐fat diet‐induced MASLD in mice. PIEZO1 negatively regulates de novo lipogenesis through activation of CaMKK2‐AMPK pathway.

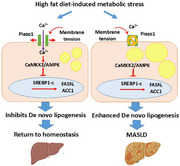

## 1 Introduction

Metabolic dysfunction‐associated steatotic liver disease (MASLD), previously referred to as non‐alcoholic fatty liver disease (NAFLD) [1, 2], is defined by the presence of steatosis in more than 5% of the liver, occurring in the absence of significant alcohol consumption or other causes of liver injury [3]. It is a heterogeneous disease that encompasses a range of pathological conditions, from simple hepatic steatosis or metabolic dysfunction‐associated steatotic liver (MASL) to metabolic dysfunction‐associated steatohepatitis (MASH), in which steatosis is accompanied by inflammation, liver injury and fibrosis [4]. It is also a common cause of end‐stage liver disease and primary liver cancer [4]. The global prevalence of MASLD has been constantly rising over the past 20 years [5]. A meta‐analysis in 2023 estimated that MASLD affects approximately 30% of global population and has become the most common cause and the most rapidly increasing disease burden of chronic liver disease around the world [6].

The liver plays a crucial role in lipid metabolism. Hepatocytes, which comprise up to 80% of the liver mass, acquire fatty acids mainly through de novo lipogenesis (DNL) and blood uptake. These fatty acids can be disposed by the hepatocytes through oxidation to produce acetyl‐CoA and ATP, or converted into triglycerides (TGs) and released into circulation as very‐low density lipoprotein (VLDL) particles [7, 8]. Under normal conditions, the uptake and utilization of fatty acids are balanced, leading to minimal storage of TGs in the hepatocytes. However, when the balance is disrupted, TGs can accumulate in the form of lipid droplets within the hepatocytes, a hallmark of steatotic livers [7, 8].

To maintain energy homeostasis, the liver employs various mechanisms to control lipid metabolism and ensure a balance between lipid acquisition and disposal. Hepatocytes are capable of detecting chemical signals from nutrients, such as glucose, amino acids, and lipids, and converting these signals to regulate de novo lipogenesis (DNL) and fatty acid oxidation (FAO) through AMPK, mTOR, and PPARs pathways [9, 10, 11, 12]. Particularly, AMPK is the primary sensor of cellular energy and a hub for lipid and carbohydrate metabolism, thus playing a central role in energy homeostasis of the body [13]. Activation of AMPK can inhibit DNL and promote FAO to maintain lipid homeostasis [9].

Recently, increasing evidence have pointed to the influence of mechanical force and mechanotransduction on energy metabolism [14, 15]. Cells can respond to the mechanical force originated from the microenvironment (such as matrix stiffness, shear force) or themselves (such as plasma membrane tension, cytoskeleton dynamics) to regulate cellular metabolism, including glycolysis [16, 17], glucose uptake [18], lipid synthesis [19], and lipophagy [20].

PIEZO1 is a mechanosensitive cation channel capable of detecting various types of mechanical force [21]. It opens when the PIEZO1‐membrane system is subjected to mechanical force and transits from curved to flattened state [22]. Once it opens, it can mediate Ca^2+^ entry into the cell and induce various biological processes through activation of Ca^2+^‐related signaling pathways. Recent studies reveal that PIEZO1 plays a role in regulating energy metabolism through mechanotransduction. PIEZO1 in pancreatic β cells can sense cell swelling to promote insulin secretion [23]. Our recent studies demonstrated that PIEZO1 in intestinal enterocytes, intestinal L cells and gastric X/A‐like cells can sense the mechanical stretching after feeding, thereby regulating nutrient absorption, GLP‐1 production and ghrelin production, and influencing postprandial blood glucose level and appetite [24, 25, 26]. In adipocyte, Wang et al. demonstrates that increased membrane tension during adipocyte maturation can activate PIEZO1, which induces adipocyte precursor differentiation and promotes adipogenesis [27]. Interestingly, when MASLD develops, hepatocytes also accumulate lipid droplets and increase in size, resembling the process of adipocyte differentiation. However, whether hepatocytes are also subjected to mechanical force and whether mechanotransduction plays a role in hepatic lipid metabolism remains unknown.

Of note, AMPK is known to be activated by Calcium/Calmodulin‐dependent Protein Kinase Kinase 2 (CaMKK2), which links it to Ca^2+^ signaling [13]. CaMKK2‐AMPK pathway has been reported to play a beneficial role in hepatic lipid metabolism. It mediates the role of Cysteine dioxygenase type 1 (Cdo1) to promotes fatty acid oxidation and mitochondrial biogenesis in hepatocytes thus conferring protective effects of exercise against MASLD [28]. CaMKK2‐AMPK has also been reported to alleviate lipid accumulation in alcohol‐induced hepatic steatosis [29] and attenuate lipotoxicity in MASLD [30]. These led us to hypothesize that PIEZO1 might regulate hepatic lipid metabolism through the Ca^2+^‐CaMKK2‐AMPK pathway. In the current study, we observed that lipid accumulation in hepatocytes led to increased membrane tension. Furthermore, PIEZO1 could sense this tension and activate the CaMKK2‐AMPK‐RAPTOR signaling pathway, thereby inhibiting de novo lipogenesis and alleviating MASLD.

## 2 Results

### 2.1 PIEZO1 is Expressed in Hepatocytes and is Downregulated in MASLD

**FIGURE 1:**
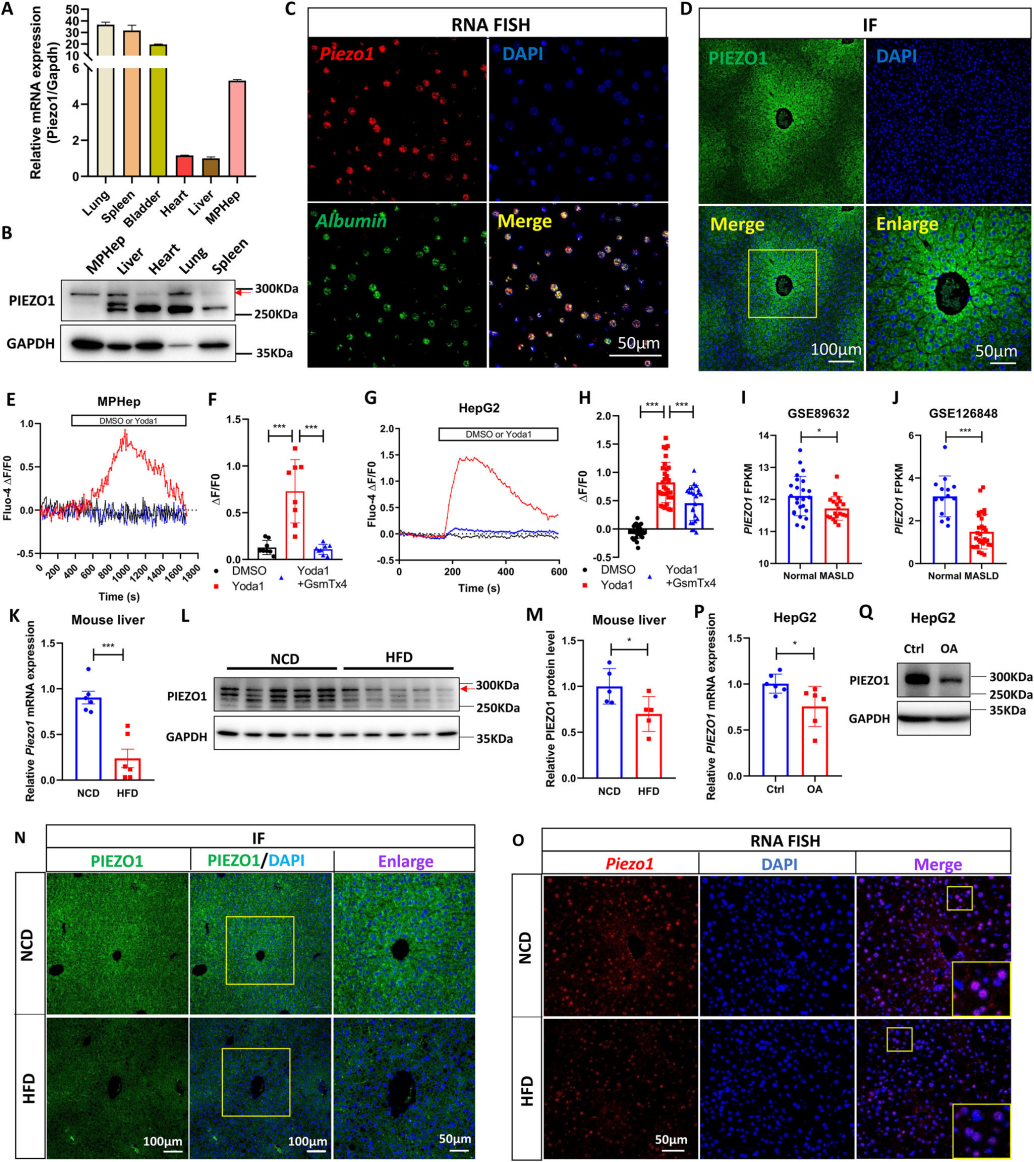
Piezo1 expression in the normal and MASLD liver. (A,B) RT‐qPCR and western blot for Piezo1 mRNA and protein in primary hepatocytes and different organs of C57BL/6 mouse. (C) FISH for Piezo1 and Albumin mRNA in C57BL/6 mouse liver. (D) Immunofluorescent staining of PIEZO1 protein in C57BL/6 mouse liver. (E) Trace of intracellular Ca^2+^ imaging of mouse primary hepatocytes (MPHep). (F) Statistics for intracellular Ca^2+^ imaging of MPHep. (G) Trace of intracellular Ca^2+^ imaging of HepG2. (H) Statistics for intracellular Ca^2+^ imaging of HepG2. Statistical data in (F) were presented as mean ± SD from *n* = 8 cells. Statistical data in (H) were presented as mean ± SD from *n* = 24∼31 cells (*n* = 31 for DMSO, *n* = 33 for Yoda1, *n* = 24 for Yoda1+GsmTx4). Statistical significance in (F&H) was determined using one‐way ANOVA followed by Tukey posthoc test ^***^ *p* < 0.001. (I,J) Analysis of PIEZO1 mRNA abundance in the livers of normal human subjects and MASLD patients with RNA‐seq datasets (GSE89632 and GSE126848). Statistical data were presented as mean ± SD from *n* = 14∼31 subjects ((I) *n* = 24 for normal, *n* = 19 for MASLD. (J) *n* = 14 for normal, *n* = 31 for MASLD). Statistical significance in (I,J) was determined using Student's *t*‐test. ^**^ *p* <0.01, ^***^ *p* <0.001. (K) RT‐qPCR for Piezo1 mRNA in the livers of C57BL/6 mice fed with normal chow diet (NCD) or high‐fat diet (HFD) for 12 weeks. (L) Western blot for PIEZO1 protein in the livers of C57BL/6 mice fed with NCD or HFD for 12 weeks. (M) Densitometry quantification of (L). Statistical data in (K,M) were presented as mean ± SD from *n* = 5∼6 biological replicates ((K) *n* = 6, (M) *n* = 5). Statistical significance in (K,M) was determined using Student's *t*‐test. ^***^ *p* <0.001. (N) Immunofluorescent staining for PIEZO1 in the livers of C57BL/6 mice fed with NCD or HFD for 12 weeks. (O) FISH for Piezo1 mRNA in the livers of C57BL/6 mice fed with NCD or HFD for 12 weeks. (P,Q) RT‐qPCR and western blot for PIEZO1 mRNA in HepG2 treated with vehicle or 250 µm OA for 24 h. Statistical data in (P) were presented as mean ± SD from *n* = 6 biological replicates. Statistical significance in (P) was determined using Student's *t*‐test. ^*^ *p* <0.05.

We first examined the expression of PIEZO1 in the liver and hepatocytes. We used the public single‐cell RNA sequencing data from human and mouse liver (GSE192742) and found that PIEZO1 is expressed in most of the cell populations in the liver, including hepatocytes, endothelial cells and immune cells (Figure S1A,B). RT‐qPCR and western blot results confirmed the expression of PIEZO1 in mouse liver and mouse hepatocytes, as well as hepatic stellate cells (Figure 1A,B; Figure S1C). RNA‐FISH (Figure 1C) and immunofluorescent (IF) staining (Figure 1D) also indicated the localization of PIEZO1 in hepatocytes. Moreover, IF staining suggested an uneven expression of Piezo1 in the liver, with higher expression in the hepatocytes near the central vein. Intracellular calcium imaging with the Ca^2+^ sensitive fluorescent probe Fluo‐4 showed that PIEZO1 activator Yoda1 induced increase in fluorescent intensity (ΔF) in both mouse primary hepatocytes (MPHep) (Figure 1E,F) and HepG2 (Figure 1G,H), indicating rise in intracellular Ca^2+^ concentration. The Yoda1‐induced intracellular Ca^2+^ increase could be inhibited by pre‐incubation of PIEZO1 blocker GsMTx‐4 in both MPHep (Figure 1E,F) and HepG2 (Figure 1G,H).

To investigate whether PIEZO1 expression is altered in MASLD, we analyzed two human MASLD liver RNAseq datasets (GSE89632 and GSE125848). Both datasets showed that MASLD patients had lower PIEZO1 expression in the liver compared to normal human controls (Figure 1I,J). In line with our finding in humans, HFD‐induced MASLD mice had lower PIEZO1 expression in the liver compared to control mice fed with normal chow diet (NCD), as shown by RT‐qPCR (Figure 1K) and western blot (Figure 1L,M). Immunofluorescence (Figure 1N) and RNA‐FISH (Figure 1O) results also showed decrease in PIEZO1 protein signal and *Piezo1* mRNA signals in the liver of HFD‐fed mice. In vitro treatment of HepG2 cells with oleic acid (OA) for 24 h also resulted in downregulation of *PIEZO1* mRNA and protein (Figure 1P,Q), which was consistent with the downregulation of PIEZO1 in the liver of MASLD patients and HFD‐induced MASLD mice.

### 2.2 Hepatocyte‐Specific PIEZO1 Loss Exacerbates HFD‐Induced MASLD

**FIGURE 2:**
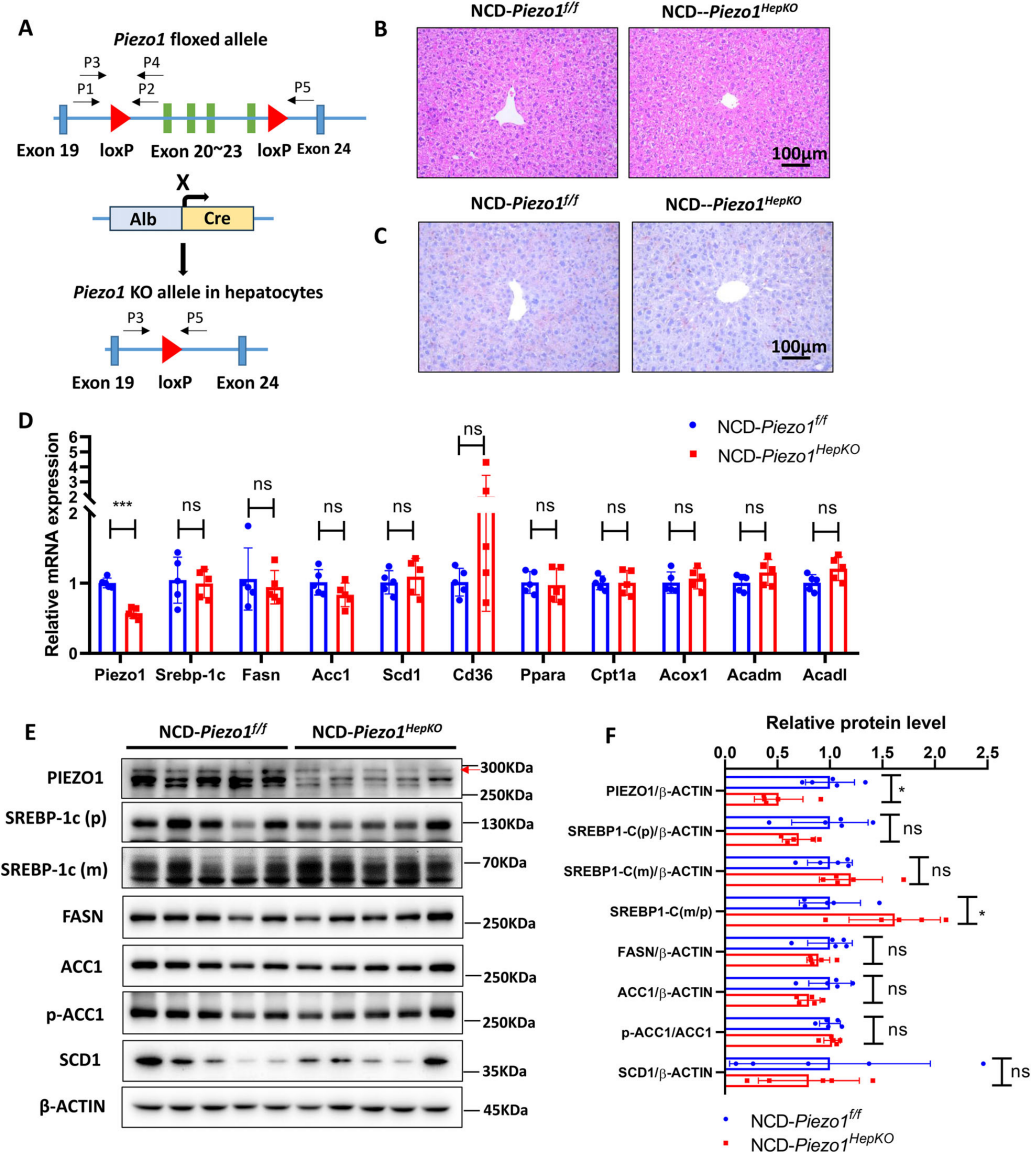
Generation and Characterization of Piezo1^HepKO^ mice. (A) Schematic description for the generation of Piezo1^HepKO^ mice. Locations of primers for genotyping are shown. (B,C) Liver H‐E staining (B) and oil‐red O staining (C) of Piezo1^f/f^ and Piezo1^HepKO^ mice fed with NCD. (D) RT‐qPCR results for genes related to DNL and FAO in the livers. (E) Western blot results for proteins related to DNL in the livers. (F) Densitometry quantification for (E). Statistical data in (D&F) were presented as mean ± SD from *n* = 5 biological replicates. Statistical significance was determined using Student's *t*‐test. ^*^ *p* <0.05, ^***^ *p* <0.001, ns: not significant.

To investigate the role of PIEZO1 in liver lipid metabolism and MASLD development, we generated mice with hepatocyte‐specific *Piezo1* deletion by crossing *Piezo1^loxP/loxP^* (*Piezo1^f/f^*) mice and *Alb‐cre* mice (Figure 2A). Genotyping result of *Piezo1^f/f^* mice and hepatocyte‐specific *Piezo1* knockout mice (*Piezo1^HepKO^*) are shown in Figure S2A. The recombination of the *Piezo1* gene in the liver of *Piezo1^HepKO^* mice was confirmed by PCR with genomic DNA (Figure S2B). RNA‐FISH also indicated decrease of *Piezo1* expression in the hepatocyte of *Piezo1^HepKO^* mice (Figure S2C). Under NCD feeding, there was no difference in liver weight (*Piezo1^f/f^ 1.049* ± 0.063 vs *Piezo1^HepKO^1.037* ± 0.126), body weight (*Piezo1^f/f^ 24.16* ± 1.86 vs *Piezo1^HepKO^ 22.68* ± 1.91) or liver‐to‐body weight ratio (*Piezo1^f/f^ 0.044* ± 0.005 vs *Piezo1^HepKO^ 0.046* ± 0.002) between *Piezo1^HepKO^* mice and their littermate *Piezo1^f/f^* controls (Figure S2D–F). Both *Piezo1^HepKO^* and *Piezo1^f/f^* mice showed normal liver morphology without significant lipid accumulation (Figure 2B,C). RT‐qPCR and western blot showed that neither DNL nor FAO genes were significantly affected by hepatocyte‐specific *Piezo1* knockout (Figure 2D–F). However, the ratio of mature SREBP‐1c (68 kDa) to SREBP‐1c precursor (120 kDa) is significantly higher in *Piezo1^HepKO^* mice (Figure 2F), implicating higher cleavage rate of SREBP‐1c protein in *Piezo1^HepKO^* mice.

**FIGURE 3:**
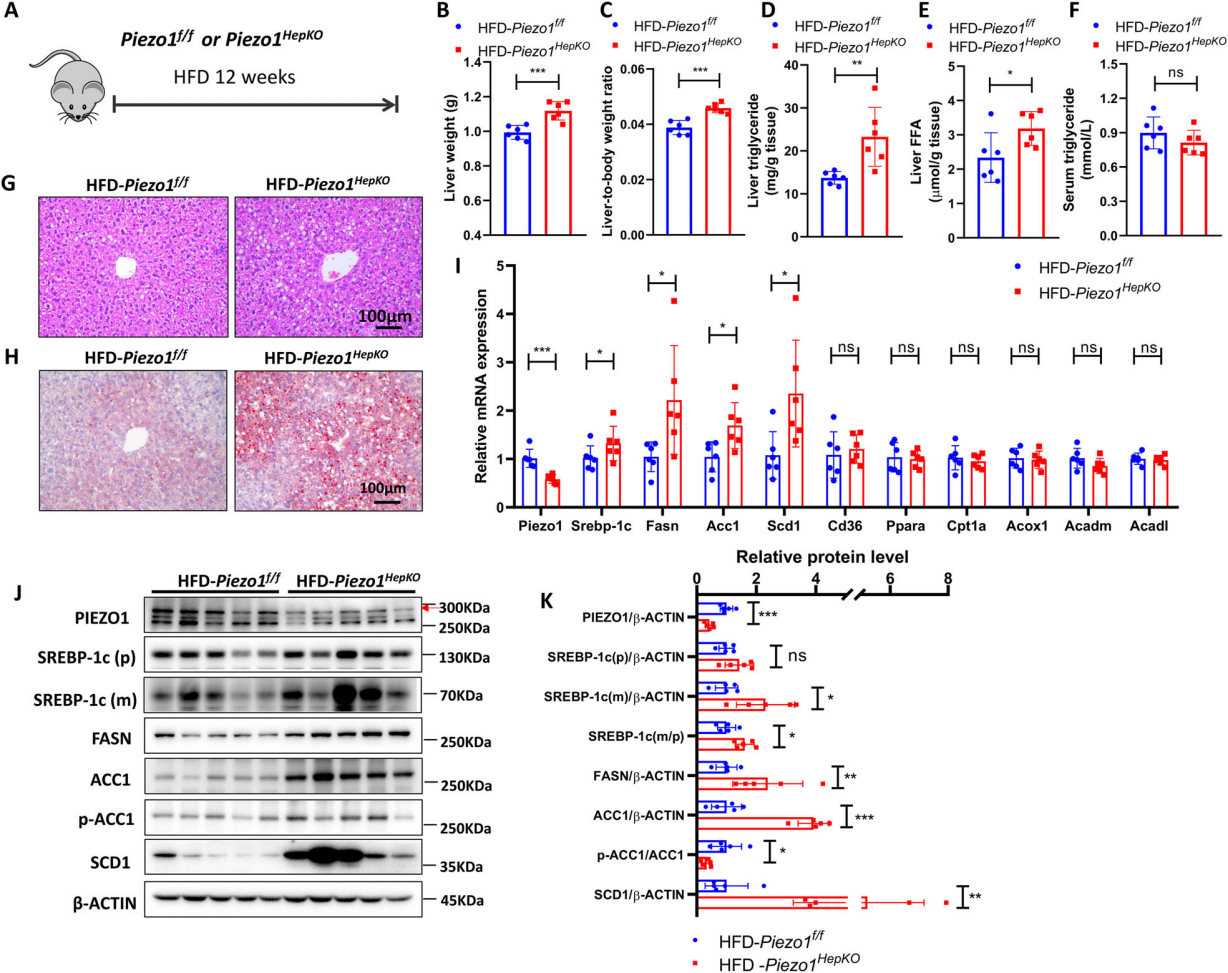
Hepatocyte‐specific Piezo1 knockout exacerbates HFD‐induced MASLD phenotype and upregulates DNL genes in the liver. (A) HFD feeding timeline. (B–E) Liver weight (B), liver‐to‐body weight ratio (C), liver triglyceride content (D), liver free fatty acid content (E) and serum triglyceride content (F) of Piezo1^f/f^ and Piezo1*^HepKO^* mice fed with HFD. (G,H) Liver H‐E staining (G) and oil‐red O staining (H). (I) RT‐qPCR results for genes related to DNL and FAO in the livers. (J) Western blot results for proteins related to DNL in the livers. (K) Densitometry quantification for (I). Statistical data were presented as mean ± SD from *n* =  5∼6 biological replicates (B‐F, I) *n* = 6, (K) *n* = 5). Statistical significance was determined using Student's *t*‐test except for SCD1 in (I) using Mann–Whiteny *U*‐test. ^*^ *p* <0.05, ^**^ *p* <0.01, ^***^ *p* <0.001, ns: not significant.

However, *Piezo1^HepKO^* mice fed with HFD for 12 weeks (Figure 3A) presented severer MASLD features, with higher liver weight (*Piezo1^f/f^ 0.993* ± 0.039 vs *Piezo1^HepKO^1.118* ± 0.053) and liver‐to‐body weight ratio (*Piezo1^f/f^ 0.039* ± 0.002 vs *Piezo1^HepKO^0.046* ± 0.002) (Figure 3B,C), increased hepatic triglyceride (*Piezo1^f/f^ 13.72* ± 1.483 vs *Piezo1^HepKO^23.27* ± 6.868) (Figure 3D) and FFA (*Piezo1^f/f^ 2.336* ± 0.723 vs *Piezo1^HepKO^3.186* ± 0.493) (Figure 3E) levels compared to littermate *Piezo1^f/f^* controls. No significant difference was observed in serum triglyceride (*Piezo1^f/f^ 0.8978* ± 0.1393 vs *Piezo1^HepKO^0.813* ± 0.1073) (Figure 3F) and total cholesterol (*Piezo1^f/f^ 17.56* ± 1.768 vs *Piezo1^HepKO^17.97* ± 1.995) (Figure S3A) between the two genotypes. Liver HE staining (Figure 3G) and oil‐red O staining (Figure 3H) indicated more lipid droplet accumulated in the liver of *Piezo1^HepKO^* mice compared to *Piezo1^f/f^* controls. The DNL genes, including Srebp‐1c, Fasn, Acc1 and Scd1, were upregulated in *Piezo1^HepKO^* mice compared to controls, while FAO genes were not significantly changed (Figure 3I–K). The ratio of mature form to precursor form of SREBP‐1c was also increased in *Piezo1^HepKO^* mice (Figure 3J,K). Phosphorylated Acc1 (p‐ACC1) to total ACC1 ratio was decreased in *Piezo1^HepKO^* mice, suggesting more active form of ACC1 in the liver of *Piezo1^HepKO^* mice (Figure 3J,K).

**FIGURE 4:**
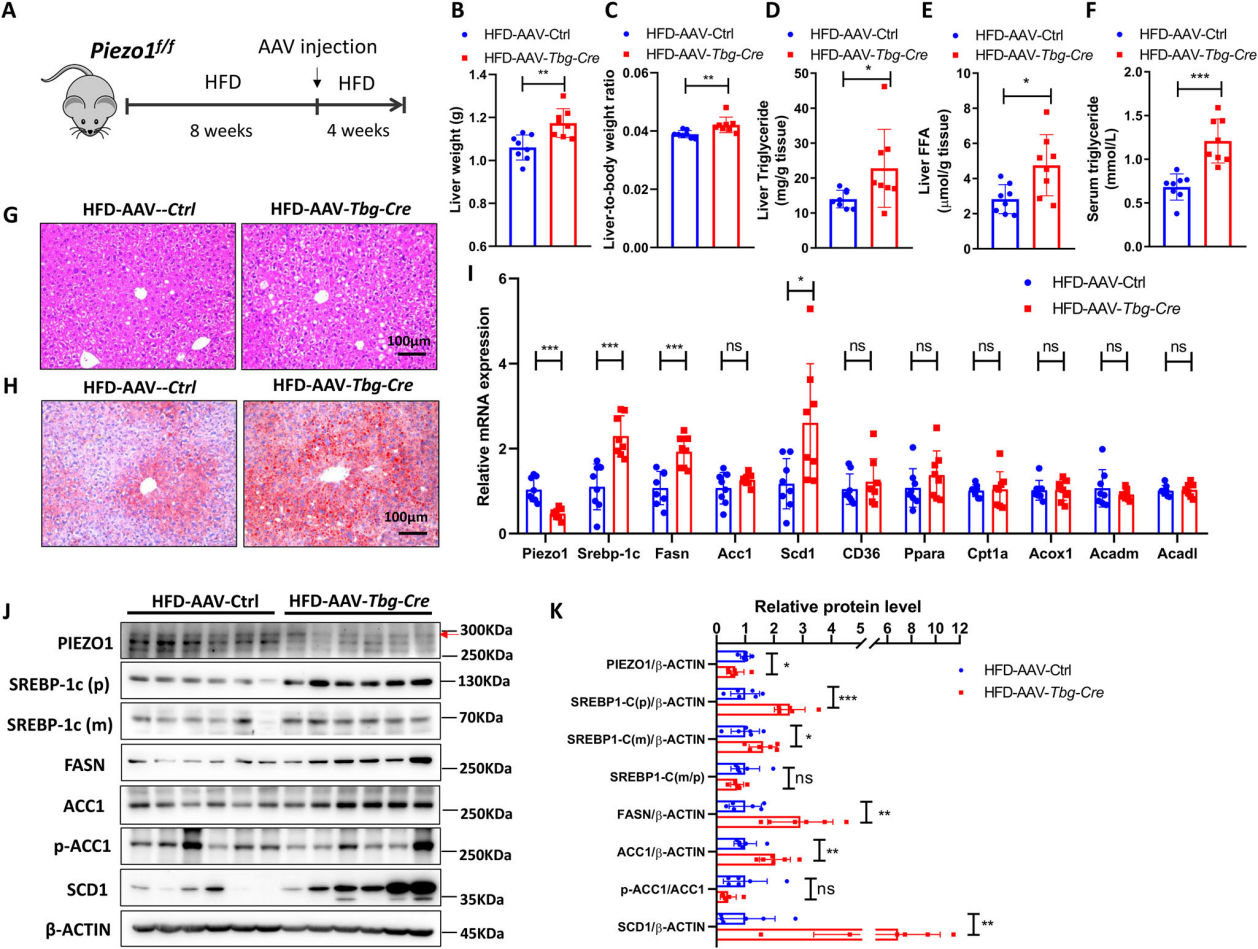
AAV‐mediated Hepatocyte‐specific Piezo1 deletion accelerates HFD‐induced MASLD phenotype and upregulates DNL genes in the liver. (A) HFD feeding and AAV injection timeline. (B–F) Liver weight (B), liver‐to‐body weight ratio (C), liver triglyceride content (D), liver free fatty acid content (E) and serum triglyceride content (F) of HFD‐fed Piezo1^f/f^ mice injected with control AAV or AAV‐Tbg‐Cre. (G–H) Liver H‐E staining (G) and oil‐red O staining (H). (I) RT‐qPCR results for genes related to DNL and FAO in the livers. (J) Western blot results for proteins related to DNL in the livers. (K) Densitometry quantification for (J). Statistical data were presented as mean ± SD from *n* = 6∼8 biological replicates ((B‐F, I) *n* = 8, (K) *n* = 6). Statistical significance was determined using Student's *t*‐test except for (D) and CD36 in (I) using Mann–Whitney *U*‐test. ^*^ *p* <0.05, ^**^ *p* <0.01, ^***^ *p* <0.001, ns: not significant.

To investigate whether loss of Piezo1 during the HFD feeding accelerate MASLD development, *Piezo1^f/f^* mice were fed with HFD for 8 weeks, followed by injection of AAV expressing hepatic‐specific promoter driven *Cre* (*Tbg‐Cre*) or control AAV through tail vein. After AAV injection, the mice were fed with HFD for additional 4 weeks (Figure 4A). In line with the finding from the constitutive *Piezo1* knockout, mice injected with AAV‐*Tbg‐Cre* showed significantly higher liver weight (*AAV‐ctrl 1.06* ± 0.058 vs *AAV‐Tbg‐cre1.174* ± 0.067) and liver‐to‐body weight ratio (*AAV‐ctrl 0.0389* ± 0.001 vs *AAV‐Tbg‐cre 0.0421* ± 0.003) (Figure 4B,C), higher hepatic triglyceride (*AAV‐ctrl 13.98* ± 2.51 vs *AAV‐Tbg‐cre 22.87* ± 11.20) (Figure 4D) and FFA (*AAV‐ctrl 2.828* ± 0.082 vs *AAV‐Tbg‐cre 4.756* ± 1.737) (Figure 4E) levels, higher serum triglyceride levels (*AAV‐ctrl 0.682* ± 0.150 vs *AAV‐Tbg‐cre* 1.208 ± 0.252) (Figure 4F) and more lipid droplet accumulation (Figure 4G,H) compared to mice injected with control AAV. No significant difference was observed in hepatic total cholesterol (*AAV‐ctrl 15.71* ± 1.70 vs *AAV‐Tbg‐cre* 17.56 ± 2.99) (Figure S3B). RT‐qPCR and western blot also indicated upregulation of DNL genes and decreased p‐ACC1/total ACC1 ratio in the liver of AAV‐*Tbg‐Cre* injected mice (Figure 4I–K). Both the constitutive knockout model and AAV‐induced knockout model suggested that loss of PIEZO1 in hepatocytes promoted DNL and exacerbated MASLD.

### 2.3 Infusion of PIEZO1 Activator Alleviates HFD‐Induced MASLD

**FIGURE 5:**
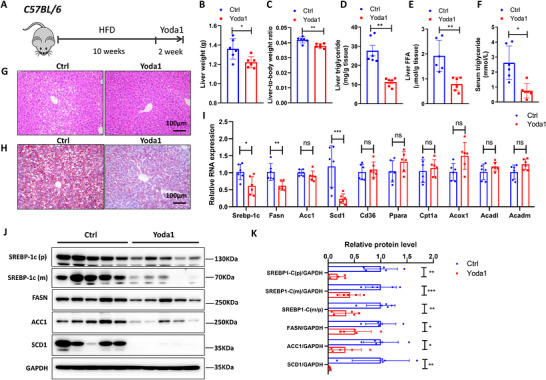
Yoda1 injection alleviates HFD‐induced MASLD phenotype and downregulates DNL genes in the livers of C57BL/6 mice. (A) HFD feeding and Yoda1 injection timeline. (B–F) Liver weight (B), liver‐to‐body weight ratio (C), liver triglyceride content (D), liver free fatty acid content (E) and serum triglyceride content (F) of HFD‐fed mice injected with vehicle control or Yoda1. (G,H) Liver H‐E staining (F) and oil‐red O staining (G). (K) RT‐qPCR results for genes related to DNL and FAO in the livers. (J) Western blot results for proteins related to DNL in the livers of HFD‐fed mice injected with vehicle control or Yoda1. (K) Densitometry quantification for (I). Statistical data were presented as mean ± SD from *n* = 5∼6 biological replicates ((B‐F, I) *n* = 6, (K) *n* = 5). Statistical significance was determined using Student's *t*‐test. ^*^ *p* <0.05, ^**^ *p* <0.01, ^***^ *p* <0.001, ns: not significant.

We next examined whether activation of PIEZO1 could alleviate MASLD. C57BL/6 mice were fed with HFD for 12 weeks. In the last two weeks of HFD feeding, mice were intraperitonially injected with PIEZO1 activator Yoda1 at 0.5 mg/kg or vehicle every day (Figure 5A). Yoda1 injected mice had lower liver weight (Ctrl 1.360 ± 0.107 vs Yoda1 1.222 ± 0.059) and liver‐to‐body weight ratio (Ctrl 0.042 ± 0.002 vs Yoda1 0.038 ± 0.001) (Figure 5B,C), lower hepatic triglyceride (Ctrl 27.70 ± 6.72 vs Yoda1 11.30 ± 2.27) (Figure 5D) and FFA (Ctrl 1.924 ± 0.627 vs Yoda1 0.789 ± 0.276) (Figure 5E) levels, lower serum triglyceride levels (Ctrl 2.616 ± 1.127 vs Yoda1 0.752 ± 0.479) (Figure 5F), lower hepatic total cholesterol (Ctrl 18.52 ± 1.62 vs Yoda1 15.02 ± 0.62) (Figure S3C) and less lipid droplet in the liver (Figure 5G,H) compared to mice injected with vehicle. DNL genes were downregulated in the liver of Yoda1 injected mice, while FAO genes were not significantly different (Figure 5I–K). It suggested that activation of PIEZO1 could ameliorate MASLD by inhibiting DNL.

### 2.4 AMPK Signaling is Impaired in *Piezo1^HepKO^* Mice Fed With HFD

AMPK is a key regulator in hepatic lipid metabolism. Activation of AMPK is known to inhibit DNL through several ways. On one hand, AMPK phosphorylates ACC1 and thus inhibits ACC1 activity. On the other hand, AMPK phosphorylates the RAPTOR, the inhibitory component of mTORC1 complex, and thus inhibits DNL.

**FIGURE 6:**
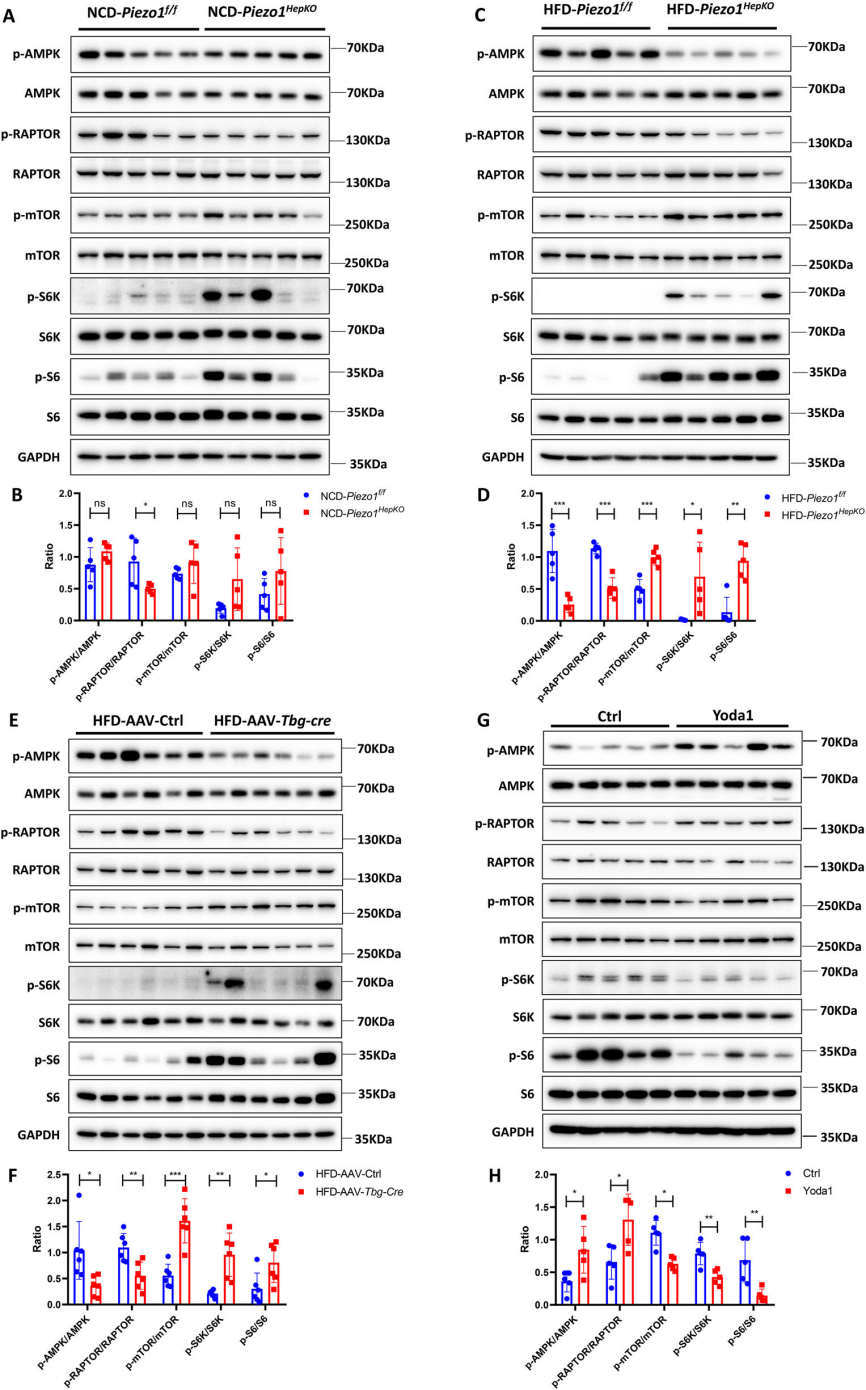
PIEZO1 enhances AMPK‐RAPTOR pathway but inhibits mTOR‐S6K‐S6 pathway in the liver. (A) Western blot results for total and phosphorylated of AMPK, RATPOR, mTOR, S6K and S6 in the livers of Piezo1^f/f^ and Piezo1^HepKO^ mice fed with NCD. (B) Densitometry quantification for (A). (C) Western blot results for total and phosphorylated of AMPK, RAPTOR, mTOR, S6K and S6 in the livers of Piezo1^f/f^ and Piezo1^HepKO^ mice fed with HFD. (D) Densitometry quantification for (C). (E) Western blot results for total and phosphorylated of AMPK, RAPTOR, mTOR, S6K and S6 in the liver of HFD‐fed Piezo1^f/f^ mice injected with control AAV or AAV‐Tbg‐Cre. (F) Densitometry quantification for (E). (G) Western blot results for total and phosphorylated of AMPK, RAPTOR, mTOR, S6K and S6 in the liver of HFD‐fed mice injected with vehicle control or Yoda1. (H) Densitometry quantification for (G). Statistical data were presented as mean ± SD from *n* =  5∼6 biological replicates ((B, D, H) *n* = 5, (F) *n* = 6). Statistical significance was determined using Student's *t*‐test except for p‐S6/S6 in (H) using Mann–Whitney *U*‐test. ^*^ *p* <0.05, ^**^ *p* <0.01, ^***^ *p* <0.001, ns: not significant.

Under NCD feeding, no difference was found in the phosphorylation of AMPK between *Piezo1^f/f^* and *Piezo1^HepKO^* mice livers. RAPTOR phosphorylation was significantly decreased in *Piezo1^HepKO^* mice livers. There is also a trend of increase in phosphorylation of mTOR and its downstream proteins S6K and S6 in *Piezo1^HepKO^* mice livers, although without statistical significance (Figure 6A,B). Under HFD feeding, significant decrease in AMPK and RAPTOR phosphorylation, but increase in mTOR, S6K and S6 were found in the liver of *Piezo1^HepKO^* mice compared to *Piezo1^f/f^* controls (Figure 6C,D). Similar results were observed in the AAV‐*Tbg‐Cre* injected mice (Figure 6E,F). In the contrary, increase in AMPK and RAPTOR phosphorylation, but decreased in mTOR, S6K and S6 phosphorylation was observed in Yoda1 injected C57BL/6 mice fed with HFD, when compared with control mice injected with PBS (Figure 6G,H). These brought us to the hypothesis that PIEZO1 might inhibit DNL through AMPK signaling pathway.

### 2.5 PIEZO1 Inhibits DNL Pathway and Enhances AMPK in Hepatocytes

**FIGURE 7:**
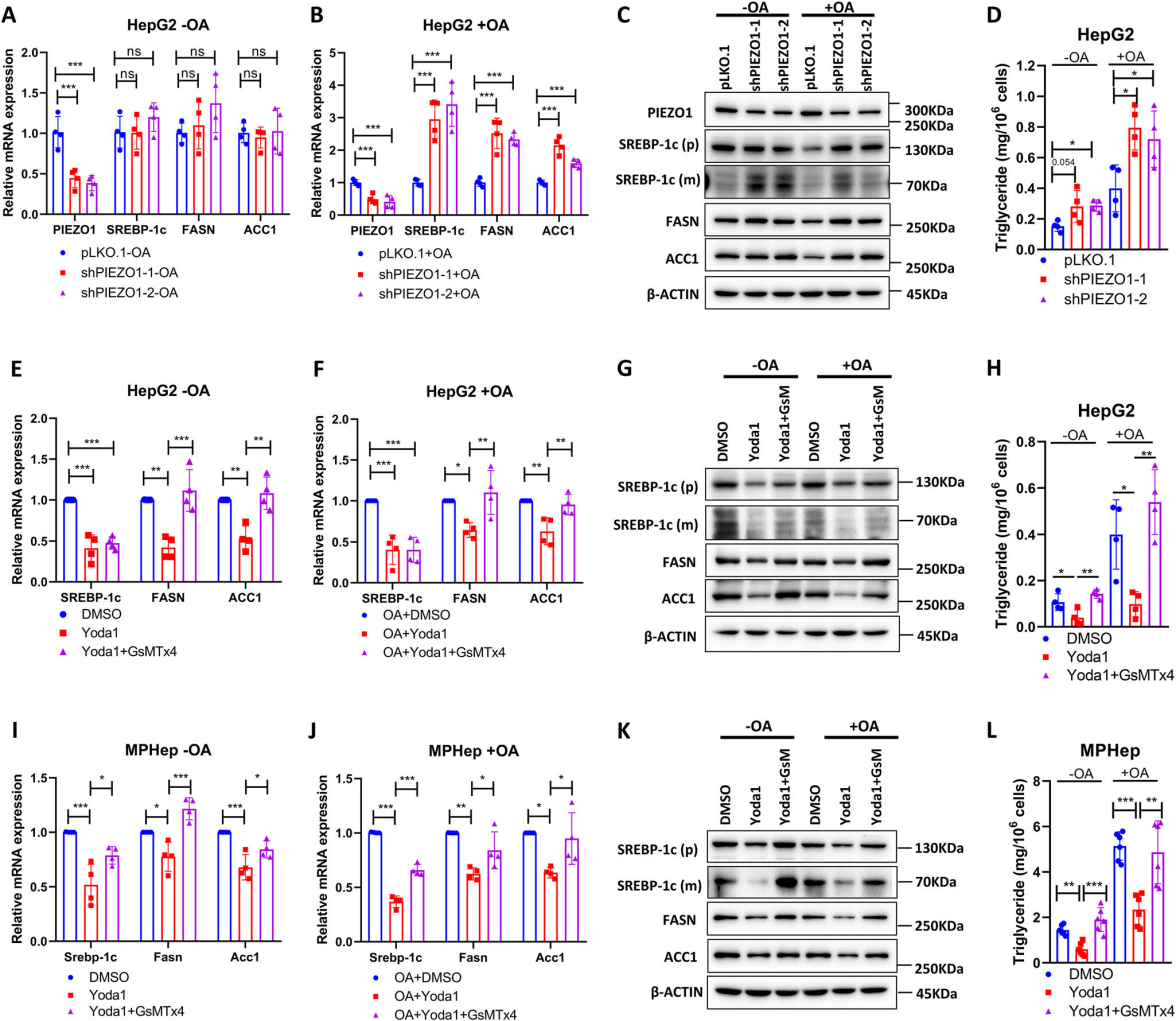
PIEZO1 inhibits DNL genes and reduced triglyceride levels in HepG2 and primary hepatocytes. (A–D) RT‐qPCR and western blot for DNL genes and cellular triglyceride measurement in control and PIEZO1 knockdown HepG2 cells in the absence or presence of 250 µm OA. (E–H) RT‐qPCR and western blot results for DNL genes and cellular triglyceride measurement in HepG2 cells treated with DMSO, Yoda1 (20 µm) or Yoda1 (20 µm) plus GsmTx4 (5 µm) in the absence or presence of 250 µm OA for 24 h. (I–L) RT‐qPCR and western blot results for DNL genes and cellular triglyceride measurement in mouse primary hepatocytes (MPHep) treated with DMSO, Yoda1 (20 µm) or Yoda1 (20 µm) plus GsmTx4 (5 µm) in the absence or presence of 250 µm OA for 24 h. Statistical data were presented as mean ± SD from *n* = 4∼6 biological replicates. Statistical significance was determined using one‐way ANOVA followed by Tukey posthoc test. ^*^ *p* <0.05, ^*^ *p* <0.01, ^***^ *p* <0.001.

Next, we validated the finding from the mouse models in hepatocyte cultures. In the absence of oleic acid (OA), knockdown of *PIEZO1* in HepG2 cells did not significantly affect mRNA expression of *SREBP‐1c*, *FASN* and *ACC1* (Figure 7A). However, in the presence of OA, *PIEZO1* knockdown led to upregulation of *SREBP‐1c*, *FASN* and *ACC1* (Figure 7B). Western blot showed that knockdown of *PIEZO1* upregulated protein levels of SREBP‐1c, FASN and ACC1 (Figure 7C). The cellular triglyceride contents were also increased in *PIEZO1* knockdown HepG2 in the presence of OA (Figure 7D; Figure S4). These recapitulated the upregulation of DNL genes in *Piezo1^HepKO^* mice and AVV‐*Tbg‐Cre* injected mice under HFD. In contrast, Yoda1 treatment for 24 h inhibited SREBP‐1c, FASN and ACC1 expression, as well as cellular triglyceride contents in both HepG2 (Figure 7E–H; Figure S5) and MPHep (Figure 7I–L; Figure S6) in the presence or absence of OA. The effect of Yoda1 could be abolished by PIEZO1 blocker GsMTx4 in both HepG2 (Figure 7E–H; Figure S5) and MPHep (Figure 7I–L; Figure S6)

### 2.6 Increase of Membrane Tension Inhibits DNL Pathway in Hepatocytes Through PIEZO1

**FIGURE 8:**
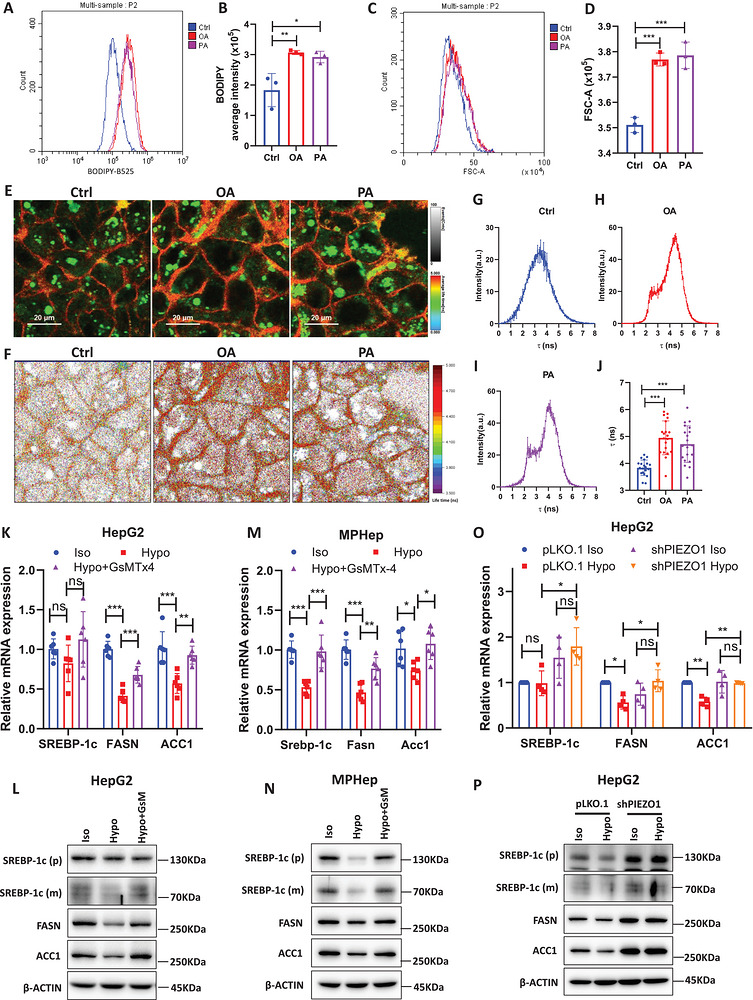
Lipid accumulation increases hepatocyte plasma membrane tension and hypotonic treatment inhibits DNL genes. (A) Distribution of BODIPY intensity in HepG2 cells detected by flow cytometry. (B) Average intensity of BODIPY in HepG2 cells with different treatments. (C) FSC distribution of HepG2 cells detected by flow cytometry. (D) Average FSC of HepG2 cells with different treatments. Statistical data were presented as mean ± SD from *n* = 3 biological replicates. Statistical significance was determined using one‐way ANOVA followed by Tukey posthoc test. ^*^ *p* <0.05, ^*^ *p* <0.01, ^***^ *p* <0.001. ^***^ *p* <0.001. (E) FLIM images of FliptR in HepG2 cells treated with vehicle, OA or PA for 24 h. (F) Heatmap for fluorescence lifetimes τvalue extracted from the FLIM images. (G–I) Distribution of fluorescence lifetimesτin HepG2 cells with different treatments. (J) Corresponding average lifetime shown in the bar chart. Statistical data were presented as mean ± SD from *n* = 20 views. Statistical significance was determined using one‐way ANOVA followed by Tukey posthoc test. ^***^ *p* <0.001. (K,L) RT‐qPCR and western blot results for DNL genes in HepG2 cells treated with isotonic solution (Iso), hypotonic solution (Hypo) or hypotonic solution with GsMTx4 (Hypo+GsMTx4) for 4 h. (M,N) RT‐qPCR and western blot results for DNL genes in mouse primary hepatocytes (MPHep) treated with isotonic solution (Iso), hypotonic solution (Hypo) or hypotonic solution with GsMTx4 (Hypo+GsMTx4) for 4 h. (O,P) RT‐qPCR and western blot results for SREBP‐1c, FASN and ACC1 in control (pLKO.1) and PIEZO1 knockdown HepG2 treated with isotonic solution (Iso) or hypotonic solution (Hypo) for 4 h. Statistical data were presented as mean ± SD from *n* = 4∼6 biological replicates ((K&M) *n* = 6, (O) *n* = 4). Statistical significance was determined using one‐way ANOVA followed by Tukey posthoc test. ^*^ *p* <0.05, ^*^ *p* <0.01, ^***^ *p* <0.001.

We hypothesized that lipid accumulation could lead to increase in plasma membrane tension of hepatocytes, which activated PIEZO1. To test this, we treated HepG2 cells with 250 µm oleic acid (OA) or 250 µm palmitic acid (PA) for 24 h to induce lipid accumulation. Flow cytometry showed that increased BODIPY intensity (Figure 8A,B) was accompanied by increased forward scatter (Figure 8C,D) in the OA or PA treated cells compared to cells treated with vehicle control, reflecting an increase in cell size when lipid droplets accumulated in the cells. To examine whether lipid accumulation affected membrane tension, we used FlipTR, a fluorescent probe that can sense lipid tension, to determine the change of plasma membrane tension of HepG2 treated with vehicle, OA or PA. We observed longer fluorescence lifetime of the probe in OA or PA treated cells compared to vehicle treated control, indicating that membrane tension increased when lipid droplet accumulated in cells (Figure 8E–J). These results suggested that accumulation of lipid could generate mechanical force in hepatocytes.

To mimic the increase of membrane tension, HepG2 and MPHep were treated with isotonic or hypotonic solution for 4 h. Hypotonic treatment, which induced cell swelling and increase in membrane tension, resulted in downregulation of FASN and ACC1 compared to isotonic control treatment (Figure 8K–N). The effect of hypotonic treatment could be inhibited GsMTx‐4. Hypotonic treatment also induced downregulation of FASN and ACC1 in control HepG2 (pLKO.1). However, this effect was abolished in the PIEZO1 knockdown HepG2 (Figure 8O,P). These results suggested that increased membrane tension downregulated DNL in hepatocytes in a PIEZO1‐dependent manner.

### 2.7 CaMKK2/AMPK Mediates the Effect of PIEZO1 on DNL

**FIGURE 9:**
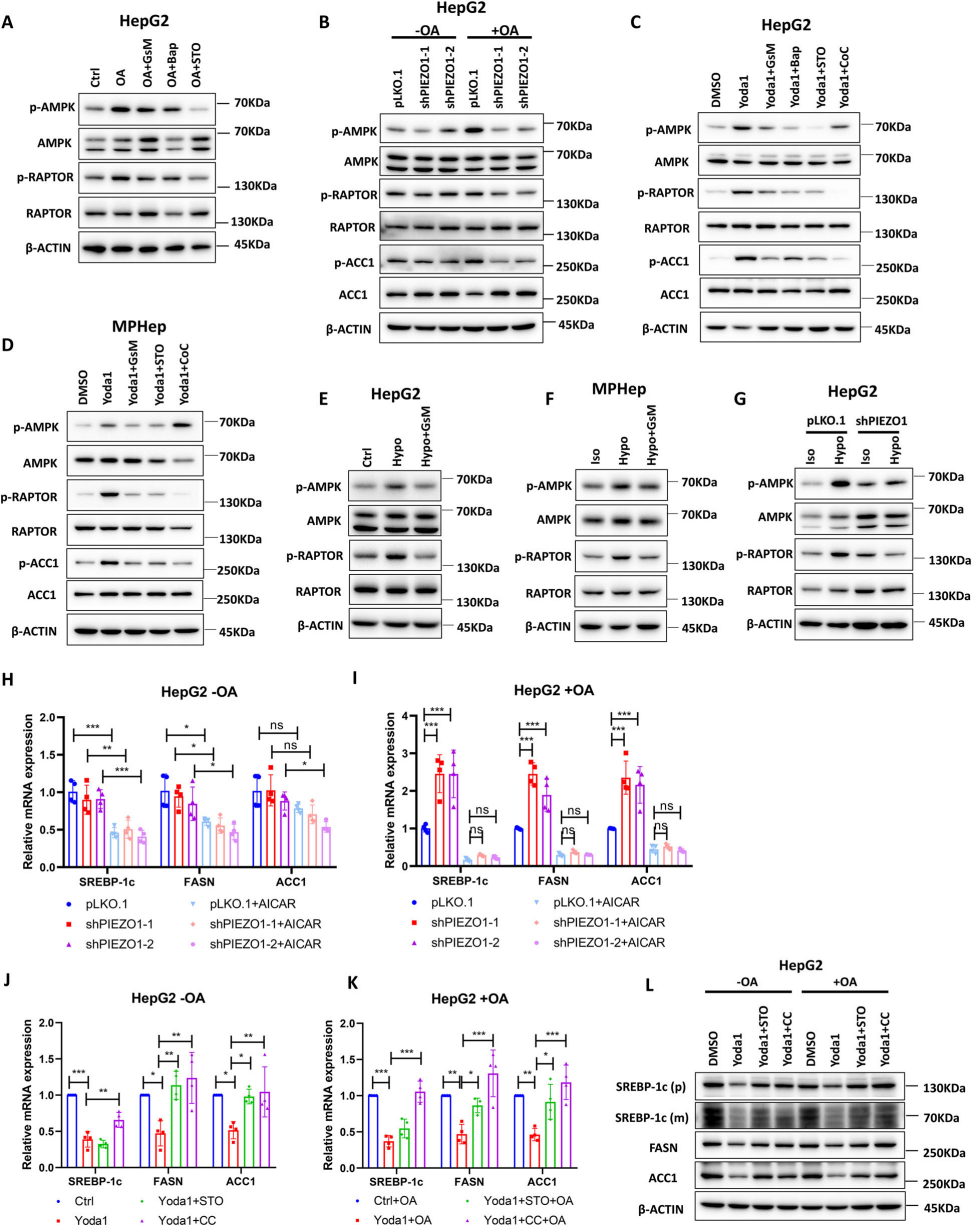
CaMKK2/AMPK mediates the effect of PIEZO1 on DNL. (A) Western blot results for phosphorylated AMPK and RAPTOR in HepG2 cells treated with vehicle, 250 µm OA, 250 µm OA plus GsmTx4 (5 µm), BAPTA‐AM (20 µm) or STO‐609 (10 µm) for 24 h. (B) Western blot results for phosphorylated AMPK, ACC1 and RAPTOR in control or PIEZO1 knockdown HepG2 cells. (C,D) Western blot results for phosphorylated AMPK, ACC1 and RAPTOR in HepG2 cells (C) or mouse primary hepatocytes (D) treated with DMSO, Yoda1 (20 µm), Yoda1 plus GsmTx4 (5 µm), Yoda1 plus BAPTA‐AM (20 µm), Yoda1 plus STO‐609 (10 µm) or Yoda1 plus Compound C (5 µm) for 30 min. (E,F) Western blot results for phosphorylated AMPK, and RAPTOR in HepG2 cells (E) or mouse primary hepatocytes (F) treated with isotonic or hypotonic solution for 4 h. (G) Western blot results for phosphorylated AMPK, and RAPTOR in control or PIEZO1 knockdown HepG2 cells treated with isotonic or hypotonic solution for 4 h. (H,I) RT‐qPCR results for DNL genes in control and PIEZO1 knockdown HepG2 cells treated with AMPK activator AICAR (1 mm) for 24 h in the absence (H) or presence (I) of OA. (J–L) RT‐qPCR and western blot results for DNL genes in HepG2 cells treated with DMSO, Yoda1 (20 µm), Yoda1 plus STO‐609 (10 µm), or Yoda1 plus Compound C (5 µm) for 24 h in the absence (J) or presence (K) of OA. Statistical data were presented as mean ± SD from *n* = 4 biological replicates. Statistical significance was determined using one‐way ANOVA followed by Tukey posthoc test. ^*^ *p* <0.05, ^*^ *p* <0.01, ^***^ *p* <0.001, ns: not significant.

We then examined whether AMPK mediated the effect of PIEZO1 by in vitro experiments. We found that OA treatment for 24 h enhanced AMPK and RAPTOR phosphorylation in HepG2, which could partially be inhibited by PIEZO1 inhibitor GsMTx4, Ca^2+^ chelator BAPTA‐AM and CaMKK2 inhibitor STO‐609 (Figure 9A), suggesting that PIEZO1 and CaMKK2 might contribute to the activation of AMPK when lipid accumulated in the cells. *PIEZO1* knockdown also led to reduced phosphorylation of AMPK, RAPTOR and ACC1 in HepG2 in the presence of OA (Figure 9B). In contrast, Yoda1 treatment elevated phosphorylation levels of AMPK, RAPTOR and ACC1 in HepG2 and MPHep (Figure 9C,D). Previous studies showed that AMPK could be phosphorylated by CaMKK2. We suspected that AMPK phosphorylation induced by PIEZO1 activation was mediated by Ca^2+^/CaMKK2. Indeed, Yoda1‐induced AMPK phosphorylation could be abolished by PIEZO1 inhibitor GsMTx4, as well as Ca^2+^ chelator BAPTA‐AM and CaMKK2 inhibitor STO‐609 (Figure 9C,D), suggesting that Yoda1‐induced AMPK phosphorylation depended on Ca^2+^/CaMKK2. Moreover, Yoda1‐induced ACC1 and RAPTOR phosphorylation could be abolished by BAPTA‐AM, STO‐609 and AMPK inhibitor Compound C (Figure 9C,D), indicating that activation of PIEZO1 stimulated AMPK‐ACC1 and AMPK‐RAPTOR pathways through Ca^2+^/CaMKK2. Similar to Yoda1 treatment, hypotonic treatment for 4 h also stimulated AMPK and RAPTOR phosphorylation in both HepG2 and MPHep, which could be abolished by PIEZO1 inhibitor GsMTx4 (Figure 9E,F). PIEZO1 knockdown also abolished the effect of hypotonic treatment on AMPK and RAPTOR phosphorylation in HepG2 (Figure 9G). The results from the hypotonic experiments supported the involvement of PIEZO1 in the membrane tension‐induced AMPK pathway activation.

To confirm the involvement of AMPK in the role of PIEZO1 in regulating DNL genes, we treated control and *PIEZO1* knockdown HepG2 with AMPK activator AICAR. In the absence of OA, AICAR downregulated *SERBP‐1c* and *FASN*, but not *ACC1* in both control and *PIEZO1* knockdown HepG2 (Figure 9H). In the presence of OA, AICAR not only downregulated *SERBP‐1c*, *FASN* and *ACC1* significantly, but also abolished the effect of *PIEZO1* knockdown on their expression (Figure 9I). In contrast, CaMKK2 inhibitor STO‐609 and AMPK inhibitor Compound C reversed the inhibitory effect of Yoda1 on SERBP‐1c, FASN and ACC1 expression (Figure 9J–L). These results indicated that the role of PIEZO1 in inhibiting DNL genes depended on CaMKK2/AMPK.

## 3 Discussion

Mounting studies have revealed the involvement of mechanical force and mechano‐sensitive proteins in regulation of metabolism. However, whether hepatocyte PIEZO1 is involved in hepatic lipid metabolism remained unclear. In the current study, we found that PIEZO1 in the hepatocytes negatively regulated lipogenesis and loss of PIEZO1 exacerbated HFD‐induced MASLD in mice, pointing out a direct role of PIEZO1 in regulating hepatic lipid metabolism.

PIEZO1 expression has been previously found in the liver and plays various roles in different liver diseases. A recent study reported that PIEZO1 in the liver macrophage enhances efferocytosis and promotes the resolution of liver fibrosis [31]. Our previous study showed that PIEZO1 in the hepatocytes protects liver from acetaminophen‐induced acute liver injury [32]. PIEZO1 also promote hepatocellular carcinoma development by promoting cell proliferation, migration and EMT [33, 34]. Although PIEZO1 are upregulated in these liver diseases, we found downregulation of liver PIEZO1 in MASLD using liver tissues from healthy subjects and MASLD patient and HFD‐induced MASLD mouse model, suggesting PIEZO1 might be a negative regulator of MASLD development. Indeed, hepatocyte‐specific deletion of *Piezo1* in mice exacerbated MASLD compared to control mice under HFD. However, it is important noted that our MASLD mouse models are at the simple steatosis stage. Further investigation is needed to determine whether hepatocyte PIEZO1 continues to play a beneficial role, and whether PIEZO1 in other liver cell populations is involved as simple steatosis progresses to MASH.

Previous studies have reported PIEZO1 exerts it role through different signaling pathways depending on tissue and disease context. In enteroendocrine cells like L cells and X/A‐like cells, PIEZO1 activates CaMKK2/CaMKIV to regulate GLP‐1 and ghrelin production [24, 25]. In bone marrow, PIEZO1 promotes bone development through NFAT‐YAP1‐ß‐catenin [35]. In liver, PIEZO1 can alleviate drug‐induced acute liver injury by enhancing Nrf2 in the hepatocytes [32]. In hepatocellular carcinoma, PIEZO1 has been reported to promote cell proliferation and migration through YAP and TGF‐β pathways [33, 34]. Here, in our present study, we found that negative regulation of lipogenesis by PIEZO1 is mediated CaMKK2‐AMPK pathway way. Recently, AMPK is shown to be mechanoresponsive. DeMali and colleagues demonstrated that AMPK could be activated in response to external mechanical forces, which facilitated the formation of a complex comprising AMPK, E‐cadherin, and liver kinase B1 (LKB1) [18]. Bertolio et al. demonstrated that AMPK mediated the inhibition of SREBP1 maturation by ECM stiffening [36]. These findings align with the mechanoregulation of the AMPK pathway by PIEZO1 identified in our study.

In a physiological scenario, PIEZO1 is activated by mechanical force. However, the type of force that activates hepatocyte PIEZO1 and regulates hepatic metabolism remains unclear. We showed that lipid accumulation led to increase in membrane tension in HepG2. It is uncertain whether this increase in membrane tension is due to an increase in cell size or changes in the phospholipid bilayer. A study by Hirata et al. demonstrated that lipid peroxidation can elevate plasma membrane tension, leading to the activation of mechanosensitive cation channels, including PIEZO1 [37]. Nonetheless, our results support the hypothesis that lipid accumulation in hepatocytes may contribute to an increase in plasma membrane tension. It is worth noted that, in addition to the membrane tension, other mechanical properties in the liver microenvironment may also change during MASLD development [38]. It is known that the stiffness of liver is increased when MASLD develops to MASH and fibrosis. Interestingly, ECM stiffening is also found to inhibit SREBP1 maturation and lipid synthesis in MCF‐10A cell line and mMSCs, and inhibit the adipogenic commitment of mMSCs [36]. A recent study revealed that increased ECM stiffness promotes inflammation and lipogenesis while suppressing lipophagy in hepatocytes in the context of MASLD, through the PIEZO1‐YAP mechanotransduction pathway [39]. This suggests that hepatocyte PIEZO1 is capable of sensing various mechanical cues within the liver. The influence of PIEZO1 may result from the combined effects of multiple mechanotransduction pathways, which could vary at different stages of MASLD. Another mechanical property that may be changed in MASLD is the nuclear deformation as the large lipid droplet formed and compress the nucleus [38]. Transcription factor YAP is found to be activated in cells with nucleus deformed by large lipid droplet [40]. It would be interesting to investigate whether and how nuclear deformation and the nuclear mechano‐sensing machinery affect liver metabolism.

What is the physiological significance of the PIEZO1‐mediated negative regulation of lipogenesis? When energy intake and expenditure are balanced, there is minimal lipid accumulation in the liver. In this scenario, PIEZO1 remains minimally activated and has little influence on lipogenesis. However, the liver can experience metabolic stress when energy intake surpasses energy expenditure. In such cases, the liver accelerates lipogenesis to store excess energy as triglycerides. With the presence of PIEZO1, the liver can sense changes in membrane tension generated by lipid accumulation, which triggers a negative feedback mechanism on lipogenesis. This process helps to limit lipid accumulation in the liver and maintain metabolic homeostasis. However, in the absence of PIEZO1, this negative feedback system is disrupted, leading to increased lipid accumulation in the liver and ultimately contributing to the development of MASLD. This may explain why, under NCD, the *Piezo1^HepKO^* mice exhibited no difference in hepatic liver accumulation compared to the control mice. However, under HFD, they developed more severe MASLD than the control mice. In a sense, PIEZO1 may act as a stress responder, detecting excess energy in the liver and initiating a negative feedback mechanism to restore homeostasis.

Our study found that injection of PIEZO1 activator Yoda1 alleviated HFD‐induced MASLD in mice, implicating PIEZO1 as a promising target for MASLD treatment. However, given that PIEZO1 is widely expressed throughout the body, systemic injection of Yoda1 may also affect organs beyond the liver. Previous studies conducted by our group have demonstrated that PIEZO1 in intestinal enterocytes, gastric X/A‐like cells or intestinal L cells indirectly influence hepatic lipid metabolism [26, 41, 42]. Systemic Yoda1 injection can reduce sugar and lipid absorption in enterocytes [26], increase GLP‐1 secretion and improve glucose homeostasis [24], while also decreasing ghrelin secretion and inhibiting appetite in C57BL/6 mice [25]. It is possible that these changes also contribute to the reduction of liver lipid content following Yoda1 treatment, which represents a limitation of our study. To determine the extent to which hepatocyte PIEZO1 contributes to the hepatic lipid‐lowering effects of Yoda1, experiments utilizing *Piezo1^HepKO^* mice may be necessary.

In summary, we reported a role of PIEZO1 in detecting membrane tension of hepatocytes and negative regulation of lipogenesis in the liver through activation of AMPK. This finding not only highlight a mechano‐regulatory mechanism governing hepatic lipid metabolism, but also offers novel insights into the development of MASLD. Moreover, PIEZO1 may serve as a promising therapeutic target, and mechanotherapy could emerge as a viable approach for treating MASLD.

## 4 Experimental Section

### 4.1 Chemical and Antibodies

Chemicals and antibodies are listed in Table S1.

### 4.2 Animals

Mice were housed under specific pathogen‐free conditions and 12 h light/12 h dark cycle, and had free access to laboratory chow and water. The animal protocols were approved by the Animal Care and Use Committee of Jinan University, and all animal experiments were conducted in accordance with relevant ethical regulations. *C57BL/6J* mice were purchased from GemPharmatech (Guangzhou, China). *Piezo1^f/f^* mice (B6.Cg‐*Piezo1^2.1Apat^*/J, Strain #: 029213) [43] and *Alb‐Cre* mice (B6.Cg‐*Speer6‐ps1^Tg(Alb‐cre)21Mgn^*/J, Strain #: 003574) [44] were purchased from Jackson Laboratory (Bar Harbor, ME, United States). To generate hepatocyte‐specific Piezo1 knockout mice (*Piezo1^HepKO^*), the *Piezo1^f/f^* mice were crossed with the *Alb‐Cre* mice. Genotype was identified by PCR with tail raw extract. Deletion of *Piezo1* gene fragment was confirmed by PCR with liver and tail DNA. Primer sequences are shown in Table S2. Only male mice were used for generating data in this study.

### 4.3 Normal Chow Diet or High‐Fat Diet Feeding

Twelve male mice aged 5 weeks C57BL/6 mice were randomly allocated to feed a normal chow diet (NCD) (Research Diet, New Brunswick, NJ, United States) or high‐fat diet (HFD) with 60 kcal%fat (Research Diet, New Brunswick, NJ, United States) for a period of 12 weeks. Six mice were used in each group. Simple randomization method was used to allocate mice to groups.

Male *Piezo1^HepKO^* mice and their littermate control *Piezo1^f/f^* mice aged 5 weeks were placed on a NCD or HFD for a period of 12 weeks. Five to six mice were used in each group. Total 22 mice were used in this experiment. After experiments, blood was collected from retro‐orbital sinus of the mice under anesthesia, then the mice were euthanatized by CO_2_.

### 4.4 High‐Fat Diet Feeding and Yoda1 Injection

Male C57BL/6J mice aged 5∼6 weeks were fed with HFD for 12 weeks. In the last two weeks of HFD feeding, mice were intraperitonially injected with PIEZO1 activator Yoda1 at 0.5 mg/kg or vehicle every day. Mice injected with Yoda1 were compared with control mice injected with vehicle. Six mice were used in each group. Total 12 mice were used in the experiment. Simple randomization method was used to allocate mice to groups. The order of treatments and measurements was random to minimize potential confounders. After experiments, blood was collected from retro‐orbital sinus of the mice under anesthesia, then the mice were euthanatized by CO_2_.

### 4.5 High‐Fat Diet Feeding and Adeno‐Associated Virus (AAV) Injection

AAV‐Tbg‐NLS‐Cre (H7521) virus or AAV‐Tbg‐GFP (H5722) virus were purchased from OBiO Technology (Shanghai, China). Five‐week‐old male *Piezo1^f/f^* mice were fed with HFD for 8 weeks, followed by tail vein injection with AAV at a dosage of 10^11^ viral genome copies (vg). After AAV injection, mice were fed with HFD for additional 4 weeks and the tissues were collected for subsequent analysis. Mice injected with AAV‐Tbg‐NLS‐Cre were compared with control mice injected with AAV‐Tbg‐GFP. Eight mice were used in each group. Total 16 mice were used in the experiment. Simple randomization method was used to allocate mice to groups. The order of injection and measurements was random to minimize potential confounders. After experiments, blood was collected from retro‐orbital sinus of the mice under anesthesia, then the mice were euthanatized by CO_2_.

### 4.6 Cell Culture

HepG2 was derived from the Cell bank of Shanghai Chinese Academy of Sciences (Shanghai, China. Identifier: CSTR:19375.09.3101HUMSCSP510. Cat. No.: SCSP‐510. RRID: CVCL_0027). 293T cells were purchased from the Meisen Cell Technology Co., Ltd. (Zhejiang, China. Cat. No.: CTCC‐001‐0188. RRID: CVCL_0063.). All the cell lines used in this study were authenticated and confirmed contamination‐free. Cell lines with passage number between 5 and 20 since the purchased passage were used in the study. Both HepG2 and 293T were cultured in DMEM (Gibco‐Thermo Fisher, Grand Island, NY, United States) supplemented with 10% fetal bovine serum (ExCell Bio, Suzhou, China), 100IU/mL penicillin and 100ug/mL streptomycin (Gibco‐Thermo Fisher, Grand Island, NY, United States).

*PIEZO1* knockdown HepG2 cell lines were established using lentivirus‐mediated RNA interference. Small hairpin RNA (shRNA) sequences targeting human PIEZO1 (shPIEZO1‐1: CTCACCAAGAAGTACAATCAT, shPIEZO1‐2: GCTGCTCTGCTACTTCATCAT) were inserted into pLKO.1‐puro vector (Addgene, Watertown, MA, United States). 293T cells were seeded in 6‐well tissue culture plate at a density of 5 × 10^5^ cells/well, cultured for 16∼18 h for attachment, then transfected with psPAX2 and pMD2G along with pLKO.1, pLKO.1‐shPIEZO1‐1 or pLKO.1‐shPIEZO1‐2 plasmids using lipofectamine 2000 reagent (Invitrogen‐Thermo Fisher, Carlsbad, CA,United States). 48 h after transfection, medium containing lentivirus was collected to infect HepG2 cells. The infected HepG2 cells were selected with 1 µg/mL puromycin. *PIEZO1* knockdown efficiency was validated by RT‐qPCR and western blot.

Primary mouse hepatocytes were obtained from 12‐week‐old mice. In brief, the mice were anesthetized by 1% barbital sodium and the livers were perfused with EGTA solution (KCl, 5.4 mm; NaCl, 137 mm; NaHCO_3_, 4.2 mm; Na_2_HPO_4_, 0.85 mm; NaH_2_PO_4_, 0.64 mm; glucose, 5 mm; HEPES, 10 mm; EGTA, 0.5 mm; pH 7.4) from the portal vein for 1∼2 min, followed by perfusion with enzymatic buffer (KCl, 5.4 mm; NaCl, 137 mm; NaHCO_3_, 4.2 mm; Na_2_HPO_4_, 0.85 mm; NaH_2_PO_4_, 0.64 mm; CaCl_2_, 3.8 mm; glucose, 5 mm; HEPES, 10 mm; Collagenase IV (Sigma, St. Louis, MO, United States), 0.025%) for 7 min. The liver was isolated and gently minced the in a sterile cell with forceps. Cells were spun, washed and cell viability was determined using Trypan blue exclusion test. Hepatocytes were plated in complete culture medium (DMEM containing 10% fetal bovine serum 100IU/mL penicillin and 100ug/mL streptomycin) and allowed to adhere for 3∼4 h. Cells were then washed twice in complete culture medium, seeded in 6‐well tissue culture plate before indicated treatments.

For RNA/protein extraction and functional assays, HepG2 or mouse primary hepatocytes were seeded in 6‐well tissue culture plate at a density of 3 × 10^5^ cells/well, cultured for 20 h for attachment, then treated with oleic acid (OA; Sigma, St. Louis, MO, United States), palmitic acid (PA; Sigma, St. Louis, MO, United States) and drugs for 24 h.

### 4.7 Hypotonic Treatment

HepG2 and mouse primary hepatocytes were seeded in 6‐well tissue culture plate at a density of 3 × 10^5^ cells/well. After attachment for 20 h, the culture medium was replaced by Krebs‐Henseleit solution (isotonic) (NaCl 118 mm, KCl 4.7 mm, MgSO_4_ 1.64 mm, KH_2_PO_4_ 1.18 mm, NaHCO_3_ 25 mm, CaCl_2_ 2.52 mm, glucose 11.1 mm, osmolarity 300∼310mosm/L) or hypotonic solution (NaCl 78 mm, KCl 4.7 mm, MgSO_4_ 1.64 mm, KH_2_PO_4_ 1.18 mm, NaHCO_3_ 25 mm, CaCl_2_ 2.52 mm, glucose 11.1 mm, osmolarity 210∼220mosm/L) and incubated in 37C, 5% CO_2_ for 4 h. After treatment, cells were collected for RNA or protein extraction.

### 4.8 Triglyceride and Free Fatty Acid Measurement

For cellular triglyceride measurement, HepG2 or mouse primary hepatocytes were seeded at 3 × 10^5^ cell/well in 6‐well tissue culture plate. After 20 h attachment, the cells were treated with OA and drugs for 24 h. The cells were then trypsinized and collected by centrifugation. Triglyceride was extracted from cultured cells and liver by n‐Heptane:isopropanol (1:1) and measured by an enzymatic reaction‐based measuring kit (Boxbio, Beijing, China). Serum triglyceride was measured by GPO‐PAP Method. Free fatty acid was extracted from liver by n‐Heptane:methanol:chloroform (24:1:25) and measured by a kit based on copper soap method (Boxbio, Beijing, China).

### 4.9 Reverse Transcription‐Quantitative Polymerase Chain Reaction (RT‐qPCR)

RNA was isolated from cells or tissues using total RNA extraction kit (Goonie, Guangzhou, China) according to the manufacturer's instruction. RNA concentration and purity was determined by NanoDrop One Spectrophotometer (Thermo Fisher Scientific, Waltham, MA, United States). 1 µg RNA was used for complementary DNA (cDNA) synthesis in 20 uL reaction volume with HiScript III 1st Strand cDNA Synthesis Kit (Vazyme, Nanjing, China). Quantitative PCR was performed with ChamQ Universal SYBR qPCR Master Mix (Vazyme, Nanjing, China) on Roche LightCycler 480 PCR facility. The primer sequences for qPCR were listed in Table S3. Relative mRNA expression was quantified by 2^(‐ΔΔCt)^ with *GAPDH* as internal control.

### 4.10 Western Blot

Tissues or cultured cells were lysed with RIPA lysis buffer (Beyotime, Shanghai, China) containing protease inhibitor cocktail (Selleck, Shanghai, China) and phosphatase inhibitor cocktail (Selleck, Shanghai, China). Protein concentration was measured by BCA protein quantification kit (Beyotime, Shanghai, China). Equal amount of protein for each sample (20 µg) was resolved on 6%–10% sodium dodecyl sulfate‐polyacrylamide gel electrophoresis (SDS‐PAGE) gels and transferred to polyvinylidene fluoride (PVDF) membrane (Merck Millipore, Darmstadt, Germany) for immunoblotting. After blocking in 5% non‐fat milk in TBST (Tris Buffered Saline containing 0.1% Tween‐20) for 1 h, the membranes were incubated in primary antibodies overnight at 4°C, followed by horseradish peroxidase (HRP) ‐conjugated secondary antibodies (Cell Signaling Technology, Danvers, MA, USA) for 1 h at room temperature. The blots were visualized by enhanced chemiluminescent reagent (NCM Biotech, Suzhou, China) and images were captured by Tanon 5200 Chemiluminescent Imaging System (Shanghai, China). Quantitative analysis of protein expression was performed using densitometric analysis of band intensities normalized to GAPHD or β‐ACTIN as loading controls by Image J.

### 4.11 BODIPY Staining and Flow Cytometry

HepG2 or mouse primary hepatocytes were seeded at 3 × 10^5^ cell/well in 6‐well tissue culture plate. After 20 h attachment, the cells were treated with OA/PA and drugs for 24 h. Cells were fixed with 4% paraformaldehyde for 10 min, and stained with 1 µm BODIPY 493/503 probe and Hoechst 33342 for 15 min (Byeotime). The images were captured by Nikon Eclipse Ni fluorescent microscopy equipped with Nikon Digital Sight 10 camera. For flow cytometry, cells were then trypsinized and washed with PBS once, then stained with 1 µm BODIPY 493/503 probe (Byeotime) in PBS for 15 min. After washed with PBS, the cells were resuspended in 500 µL PBS. FSC, SSC and BODIPY signal were detected by flow cytometry (Beckman Coulte Cyto Flex LX).

### 4.12 Fluorescence Lifetime Imaging Microscopy (FLIM)

HepG2 cells were seeded into glass bottom confocal imaging dishes at a density around 5 × 10^4^/cm^2^ and treated with vehicle, 250 µm OA or 250 µm PA for 24 h. Prior to imaging, cells were incubated with 1 µm Flipper‐TR fluorescent cell membrane tension probe (Spirochrome, Switzerland) for 15∼20 min at 37°C. For FLIM acquisition, samples were transferred to a temperature‐controlled microscope stage maintained at 37°C. Imaging was performed using OLYMPUS IX73 confocal microscope equipped with a 100× oil‐immersion objective (NA 1.49) and a 20 MHz pulsed laser at 485 nm excitation wavelength. Fluorescence decay kinetics were recorded using time‐correlated single‐photon counting (TCSPC) with a 485/20 nm emission filter. At least 10 regions of interest (ROIs) per cell were analyzed to account for spatial heterogeneity in membrane tension. Data analysis was performed using bi‐exponential fitting algorithms (SPT‐FLIM toolbox) to resolve dual fluorescence lifetimes (τ_1_ and τ_2_) of Flipper‐TR, as previously validated. The longer lifetime component (τ_1_) was used to quantify membrane tension based on the established linear correlation between τ_1_ and tension states [45].

### 4.13 Intracellular Calcium Imaging

HepG2 or mouse primary hepatocytes were seeded in 2 cm diameter glass bottom dishes for confocal imaging at a density of 1 × 10^5^ cells/dish and culture for 20 h for attachment before imaging. On the day of imaging, cells were washed twice with HBSS with Ca^2+^ and Mg^2+^ (KCl, 5.3 mm; KH_2_PO_4_, 0.44 mm; NaCl, 138 mm; NaHCO_3_, 4.2 mm; CaCl_2_, 1.26 mm; MgCl_2_, 0.49 mm; MgSO_4_, 0.41 mm; Na_2_HPO_4_, 0.34 mm; glucose, 5.6 mm;), and incubated with 3 µm fluo‐4 AM (Invitrogen‐Thermo Fisher, Carlsbad, CA,United States) and 0.1% Pluronic F‐127 (Invitrogen‐Thermo Fisher, Carlsbad, CA,United States) in HBSS for 1 h at 37°C. The free probes were washed out with HBSS. The cells were excited with 488‐nm laser of confocal laser scanning microscopy (Zeiss, LSM880) and images were collected from 520‐nm emitted light with a digital camera. The change of intracellular calcium at a specific timepoint was reflected by the ratio between the change of fluorescent intensity to the initial fluorescent intensity (ΔF/F0).

### 4.14 RNA Fluorescence In Situ Hybridization (RNA FISH)

Paraffin sections were dewaxed and rehydrated. Following pH 6.0 antigen retrieval in citrate buffer, the sections were incubated with Proteinase K (5 µg/mL) at 37°C for 15 min. Subsequently, the sections were hybridized with the probes specific for mouse *Piezo1* (probe sequences: CTGCAGGTGGTTCTGGATATAGCCC, AAGAAGCAGATCTCCAGCCCGAAT, GCCATGGATAGTCAATGCACAGTGC) and mouse *Albumin* (probe sequences: CAGACTCATCGGCAACACACGTCT, TTGTCAGCCTCTGCACAACACTGG, ACATGTACTTGGCAAGTTCCGCCC, CGTAGCATGCGGGAGGATTGGCTT, CAAGGTTTGGACCCTCAGTCGAGA) overnight in a temperature‐controlled chamber at 40°C. After washing with SSC buffers, the sections were hybridized with pre‐warmed branched probes at 40°C for 45 min. Following another round of washing with SSC buffers, the sections were hybridized with the signal probe at 42°C for 3 h. Images were captured using fluorescent microscope.

### 4.15 Immunofluorescence

Liver tissues were fixed with 4% paraformaldehyde (Servicebio, Wuhan, China) overnight, dehydrated, embedded in paraffin blocks and cut into 4 µm sections. The sections were dewaxed and rehydrated, followed by antigen retrieval in pH 9.0 Tris‐EDTA retrieval buffer (Proteintech, Wuhan, China). Then the sections were blocked with 5% normal goat serum (Jackson ImmunoResearch, West Grove, PA, United States) in PBS for 1 h at room temperature and incubated with primary antibodies at 4°C overnight. On the next day, the sections were washed 3 times in PBS and incubated with fluorescent labeled secondary antibody for 1 h at room temperature. After three washes in PBS, the sections were mounted with ProLong Gold Antifade Mountant with DAPI (Invitrogen‐Thermo Fisher, Carlsbad, CA,United States). The images were captured by Nikon Eclipse Ni fluorescent microscopy equipped with Nikon Digital Sight 10 camera.

### 4.16 Oil‐Red O Staining

Liver tissues were fixed with 4% paraformaldehyde overnight, then embedded in Tissue‐Tek O.C.T. compound (Sakura, Torrance, CA, United States) and frozen. OCT‐embedded tissue blocks were cut into 10 µm sections. The sections were stained with oil‐red O (Sigma, St. Louis, MO, United States) working solution for 10 min and immersed into 60% isopropanol solution for 3 s for twice, followed by two washes in water for 10 s each time. Finally, the sections were counterstained with hematoxylin.

### 4.17 Statistical Analysis

For animal experiments, power analysis was performed with PASS16 to determine the n number, assuming a 1.5‐fold change between two groups and using α = 0.05, Power = 80%. For animal experiments, *n* = 5∼8 biological replicates were included. For cellular experiments, *n* = 3∼6 biological replicates were included. All data were presented as mean ± standard deviation (SD). Graphs were generated by GraphPad Prism version 8 (GraphPad Software Inc., La Jolla, CA, United States). Normality of the data was tested by Shapiro–Wilk test. For data that passed normality test, statistical differences between two groups were evaluated by two‐sided Student's *t*‐test and statistical differences among three or more groups were evaluated by two‐sided one‐way ANOVA followed by Tukey posthoc test. For data that did not pass normality test, statistical differences between two groups were evaluated by two‐sided Mann–Whitney *U*‐test. *p*‐values <0.05 were considered statistically significant. No data was excluded.

## Author Contributions

H.C., G.X., Q.Z., and J.G. contributed to conceptualization and design; H.C., G.X., and J.G. developed the methodology; H.C., Q.W., K.Y., Q.L., X.P., Z.G., X.Z., Y.W., T.T., S.X., Y.C., and X.T. performed the investigation; H.C., Q.W., Q.L., and Z.G. carried out visualization; H.C. and G.X. supervised the work; and H.C. wrote the original draft and conducted the review and editing.

## Funding

This study was supported by the National Key Research and Development Program of China (2024YFA1107200), the National Natural Science Foundation of China (No. 82470904, 82570987, 82425010, 12372317), Natural Science Foundation of Guangdong Province, China (No. 2023A1515012686 and No. 2024A1515012988), Fostering Fund of The Third Affiliated Hospital of Sun Yat‐Sen University for NSFC Program (No. 2022GZRPYMS01), Hospital University United Fund of The Second Affiliated Hospital, School of Medicine, The Chinese University of Hong Kong, Shenzhen (HUUF‐ZD‐202302).

## Ethics Approval

The animal protocols were approved by the Animal Care and Use Committee of Jinan University (IACUC‐20241119‐01), and all animal experiments were conducted in accordance with relevant ethical regulations.

## Conflicts of Interest

The authors declare no conflicts of interest.

## Supporting information




**Supporting File**: advs75082‐sup‐0001‐Data.zip.

## Data Availability

The data that support the findings of this study are available from the corresponding author upon reasonable request.
