# Supplementary figures and images for: Hepatocyte PIEZO1 Negatively Regulates Lipogenesis and Ameliorates MASLD by Sensing Membrane Tension and Activating AMPK
Source: Adv Sci (Weinh). 2026 Apr 3;13(34):e15847. doi: 10.1002/advs.202515847 (PMC13285159; doi:10.1002/advs.202515847)

Raw images of Fig.6 C

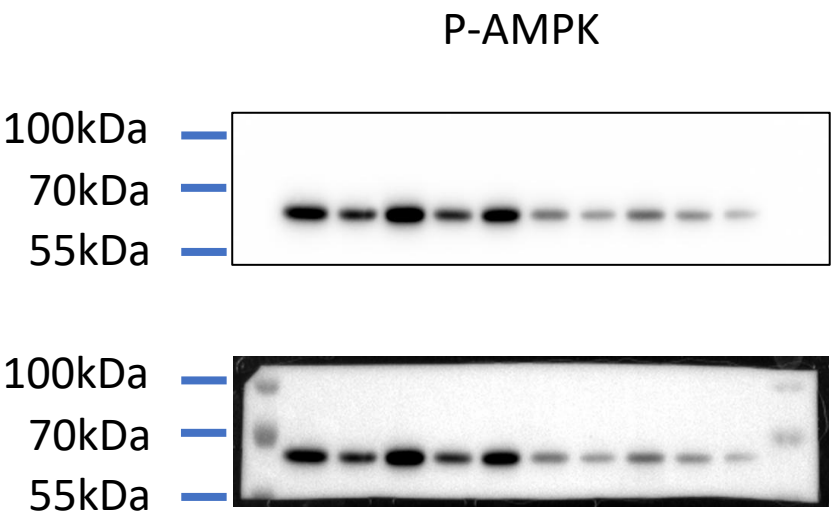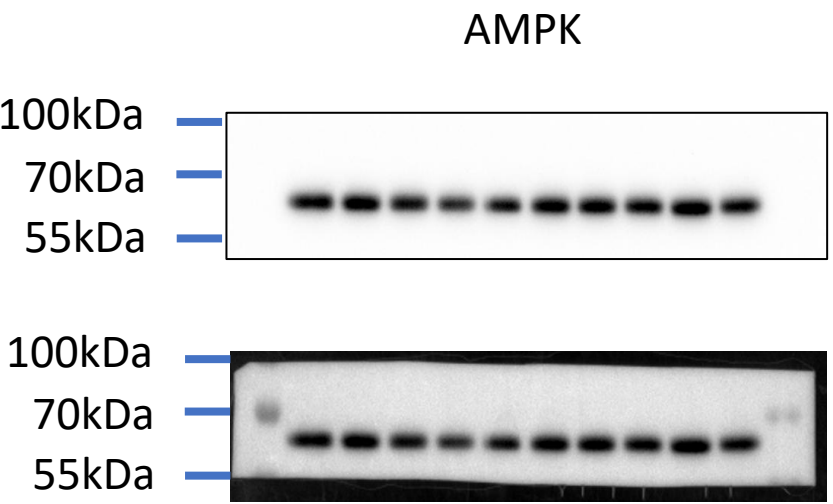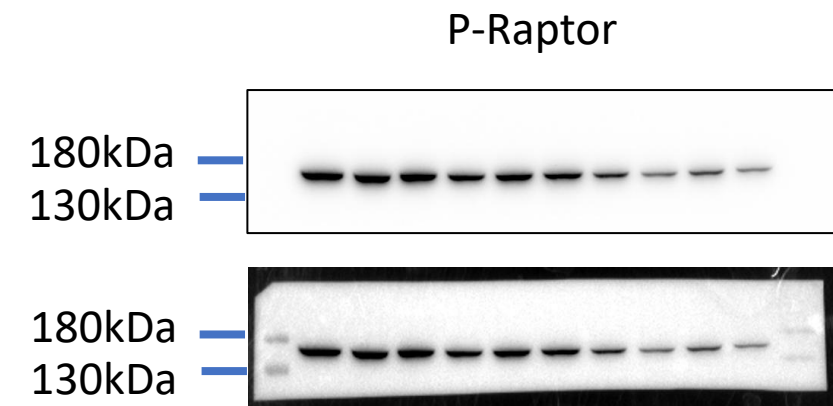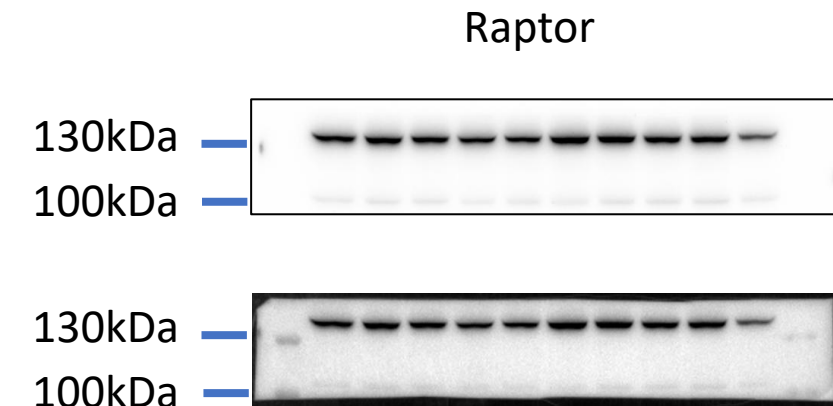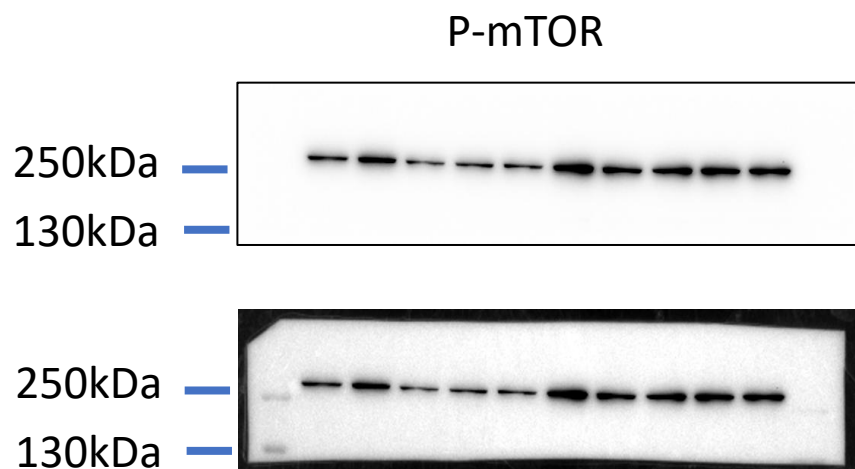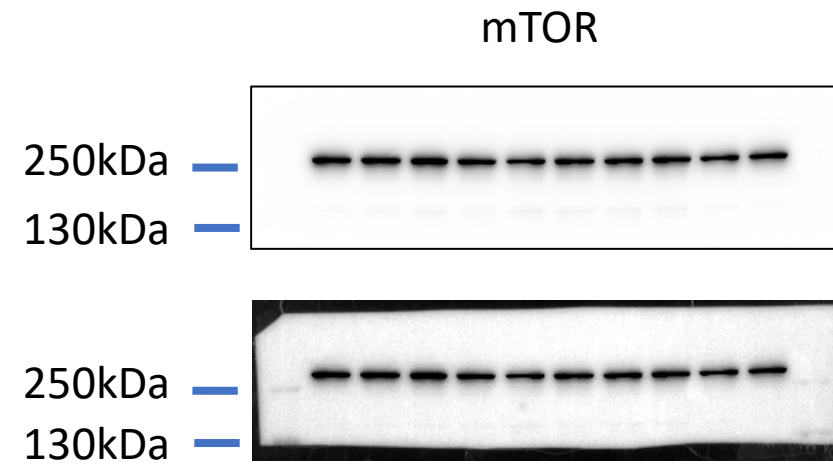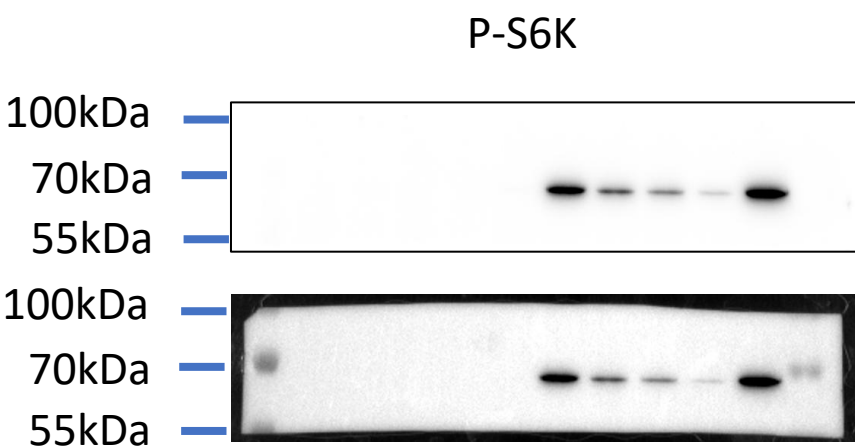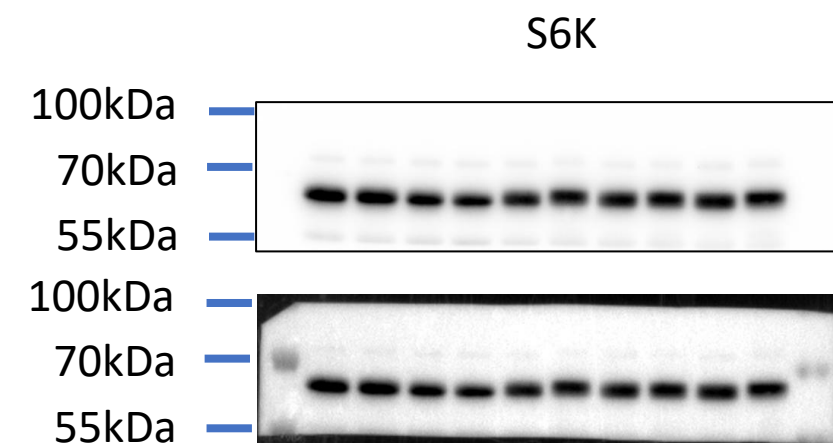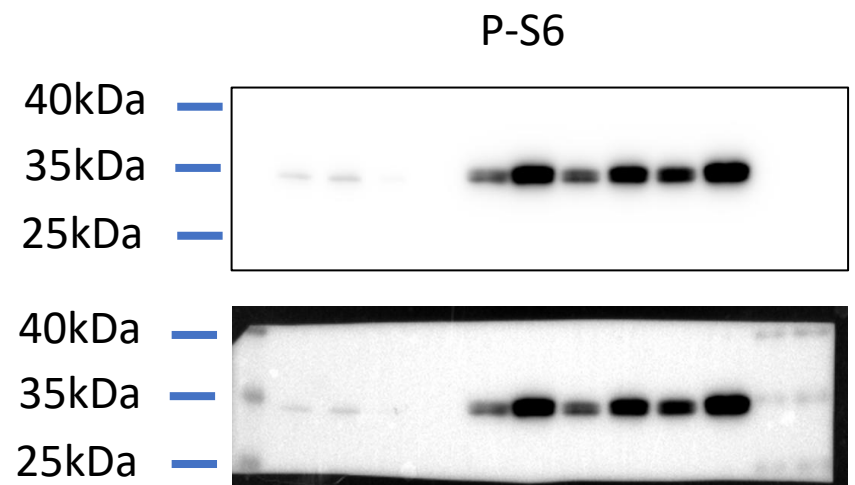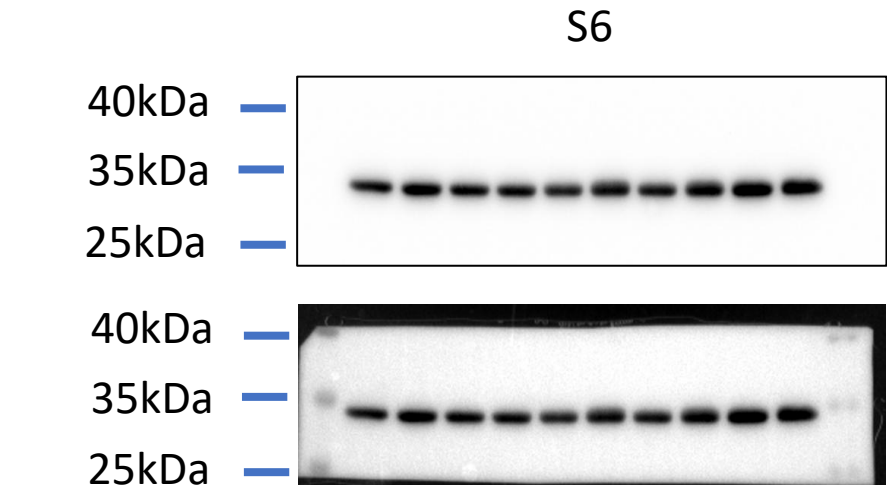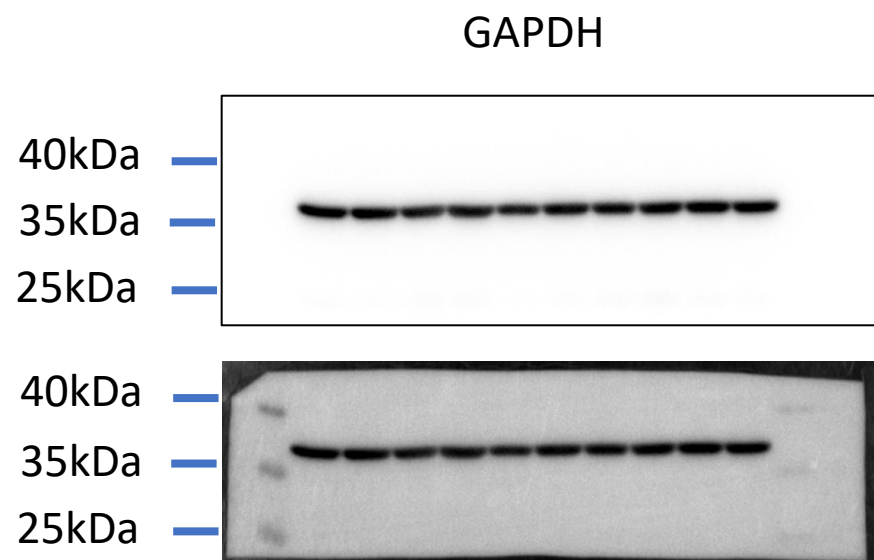

Supplement: Supplementary file 1 — Supporting File: advs75082‐sup‐0001‐Data.zip. [file ADVS-13-e15847-s001.zip › advs75082-sup-0001-Data/Fig.6C raw data of WB merge.pdf]
